# Theoretical studies on donor–acceptor based macrocycles for organic solar cell applications

**DOI:** 10.1038/s41598-022-19348-5

**Published:** 2022-09-03

**Authors:** Sheik Haseena, Mahesh Kumar Ravva

**Affiliations:** Department of Chemistry, SRM University-AP, Guntur, Andhra Pradesh 522240 India

**Keywords:** Computational chemistry, Density functional theory

## Abstract

We have designed a series of new conjugated donor–acceptor-based macrocyclic molecules using state-of-the-art computational methods. An alternating array of donors and acceptor moieties in these macrocycle molecules are considered to tune the electronic and optical properties. The geometrical, electronic, and optical properties of newly designed macrocyclic molecules are fully explored using various DFT methods. Five conjugated macrocycles of different sizes are designed considering various donor and acceptor units. The selected donor and acceptors, viz., thiophene (PT), benzodithiophene (BDT), dithienobenzodithiophene (DTBDT), diketopyrrolopyrrole (DPP), and benzothiazole (BT), are frequently found in high performing conjugated polymer for different organic electronic applications. To fully assess the potential of these designed macrocyclic derivatives, analyses of frontier molecular orbital energies, excited state energies, energy difference between singlet–triplet states, exciton binding energies, rate constants related to charge transfer at the donor–acceptor interfaces, and electron mobilities have been carried out. We found significant structural and electronic properties changes between cyclic compounds and their linear counterparts. Overall, the cyclic conjugated D–A macrocycles’ promising electronic and optical properties suggest that these molecules can be used to replace linear polymer molecules with cyclic conjugated oligomers.

## Introduction

Macrocyclic π-conjugated molecules have recently emerged as a new family of materials due to their unique structural and electronic properties^1–5^. Macrocyclic π-conjugated molecules have several advantages: controlled structure, delocalized π-space, well-defined cavity to host electronically active materials, better intermolecular arrangement, three-dimensional charge transport, and lower aggregation^6,7^. The design and development of new cyclic π-conjugated compounds, such as cyclothiophenes, cyclo-para-phenylenes (CPP), and cyclo-meta-phenylenes, have opened up new avenues for research in macrocyclic compounds^2,8,9^. Numerous reports demonstrated the applications of these materials in organic solar cells (OSCs), organic field-effect transistors (OFETs), organic light-emitting diodes (OLEDs), sensors, and photodetectors^10–14^. The choice of donor/acceptor building units and linkers dictates macrocycles' electronic and optical properties similar to conventional conjugated donor–acceptor polymers. Effective synthetic chemistry strategies have been utilized to synthesize various types of macrocycles, which are composed of triphenylamine, carbazole, thiophene, furan, acetylene, perylenediimides, etc^4,6,15,16^.

Non-fullerene acceptors with acceptor–donor–acceptor (A–D–A) units are efficient topologies for tuning HOMO–LUMO energy levels, altering the band gap, and improving absorption strength. The above-mentioned building blocks have exceptionally enhanced the performance of OSCs power conversion efficiency (PCE) of 18%)^17–21^. PCE determines how much incident light is converted to electrical energy. It is measured as the ratio of electrical output to incident solar power and expressed as PCE = (J*sc* x V*oc* x FF)/P_*in*_, where J*sc* denotes short circuit current, V*oc* stands for open circuit voltage, FF stands for fill factor, and P_*in*_ represents the power of an incident ray of light.

Thus, the design and development of novel NFA molecules have huge application potential. Particularly, the investigations on the NFA molecules comprising donor–acceptor-based π-conjugated macrocyclic molecules have paramount importance in the development of the new class of materials^22,23^. Li et al. have synthesized diketopyrrolopyrrole (DPP) based donor–acceptor macrocyclic conjugated molecules, and these materials were used as electron acceptors in OSCs. Various attributes of these macrocyclic molecules include (i) the three-dimensional shape, (ii) conjugated π-electronic delocalization, and (iii) low-energy unoccupied molecular orbital (LUMO), rendering these macrocycles as pseudo fullerenes^6^. The experimental studies of cyclo-phenylene-thienylenes (CPT) showed that the LUMO energy decreases with the increase of ring size. However, there was no substantial change in the HOMO energy^4^. At the same time, red-shift in absorption maxima and considerable blue shift in fluorescence maxima in CPT have been noticed. Similarly, Zhang et al. reported triphenylamine and benzothiadiazole-based donor–acceptor conjugated macrocycle^2^. Further, these authors have fabricated solar cells using C_60_ derivatives as acceptor units. Both scanning tunneling microscopy (STM) and density functional theory (DFT) based calculations have been employed to understand the morphology and electronic structure. A host–guest architecture of fullerene acceptors encapsulated inside cycloparaphenylene (CPP) and its derivatives have also been reported. It is found that the solid-state packing directly impacts morphology and charge transfer. The predicted PCE using microscopic charge transport parameters and a time-domain drift–diffusion model is found to be 9%^24^.

**Figure 1:**
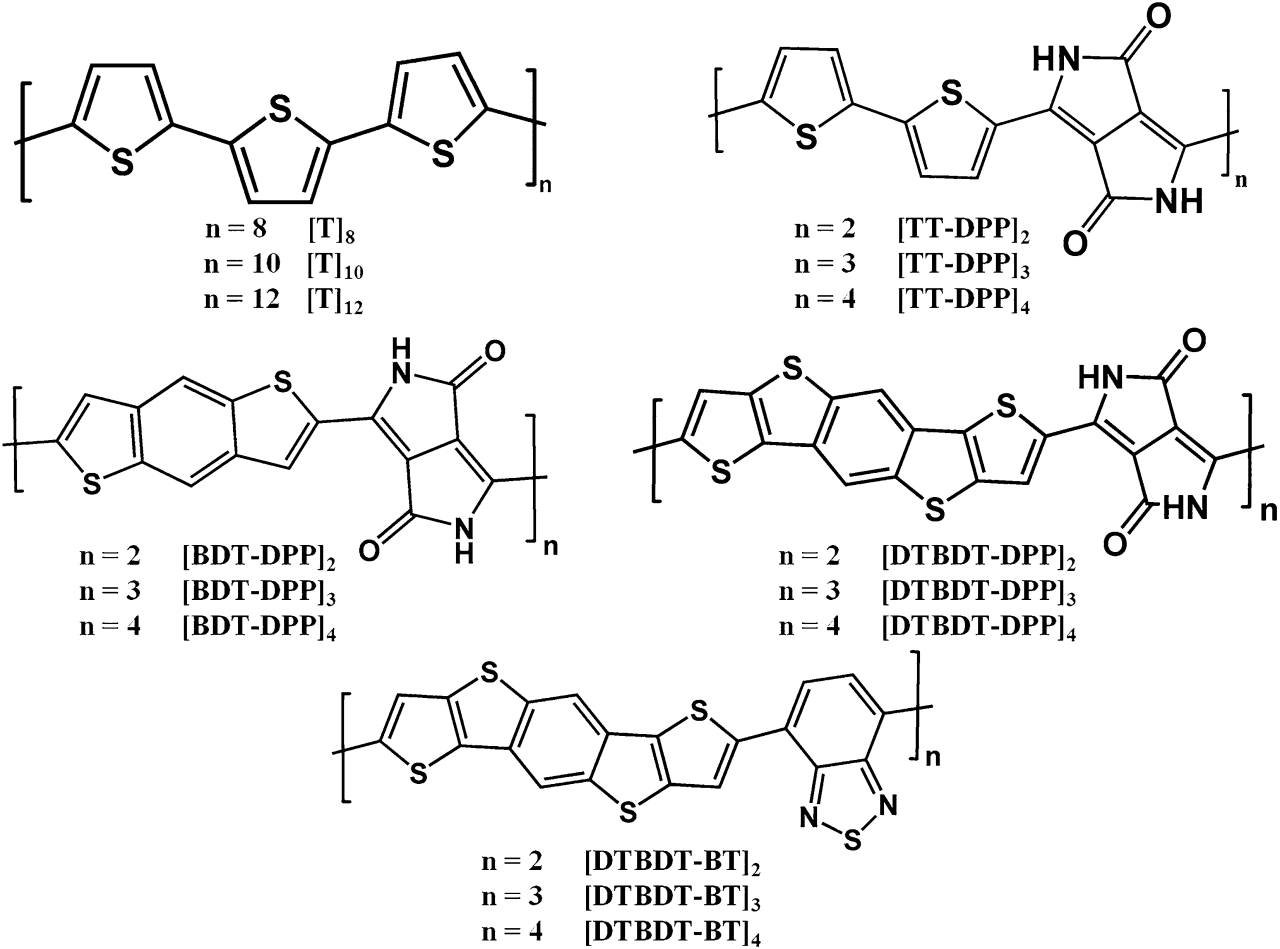
Chemical structures of donor–acceptor based conjugated systems considered in this study.

In view of the significance of macrocyclic π-conjugated materials, we have designed and developed macrocyclic compounds employing electronic structure theory. Since thiophene-based molecules have received widespread attention from researchers in the development of materials for organic electronic materials, polythiophenes-based macrocyclic π-conjugated compounds have been considered. Furthermore, the thiophene unit has been systematically replaced with different donor and acceptor units to develop various new conjugated donor–acceptor macrocycles. Density functional theory methods are used to evaluate the electronic and optical properties, viz*.,* energy levels, absorption spectra, and charge transport properties. Appropriate combinations of donor and acceptor units have been optimized by considering geometrical and electronic factors. In this context, we have taken into consideration of various critical parameters such as variation of molecule size, shape, length, orientation, and self-assembling nature, which in turn influence the nature of π-conjugation. In addition, corresponding linear counterparts have been studied to compare changes in the electronic and optical properties upon cyclization. Attempts have been made to compare experimental findings wherever possible. Overall, the findings from this study would pave the way for the development of a novel class of compounds for organic solar cell applications. The schematic representation of models considered in this study is shown in Fig. 1. As described earlier, in order to understand the impact of the size of the macrocycle on the electronic and optical properties, three different ring sizes are considered. Cyclic and linear oligothiophenes with 8, 10, and 12 repetitive thiophene units (C[PT]_n_ and L[PT]_n_, where n = 8, 10, and 12, respectively) were considered^25^. As shown in Fig. 1, four donor–acceptor-based macrocycles (C[TT-DPP]_n_, C[BDT-DPP]_n_, C[DTBDT-DPP]_n_, and C[DTBDT-BT]_n_) are designed by considering three donor units such as bithiophene (TT), benzodithiophene (BDT), dithienobenzodithiophene (DTBDT); and two acceptor units such as diketopyrrolopyrrole (DPP), and benzothiazole (BT)^6,26–31^. In order to understand the impact of cyclization, linear counterparts are also considered (L[TT-DPP]_n_, L[BDT-DPP]_n_, L[DTBDT-DPP]_n_, L[DTBDT-BT]_n_). Again, in each case, we considered two, three, and four repeating units (n = 2, 3, and 4) for both cyclic and linear molecules.

## Results and discussion

### Geometry analysis

**Figure 2:**
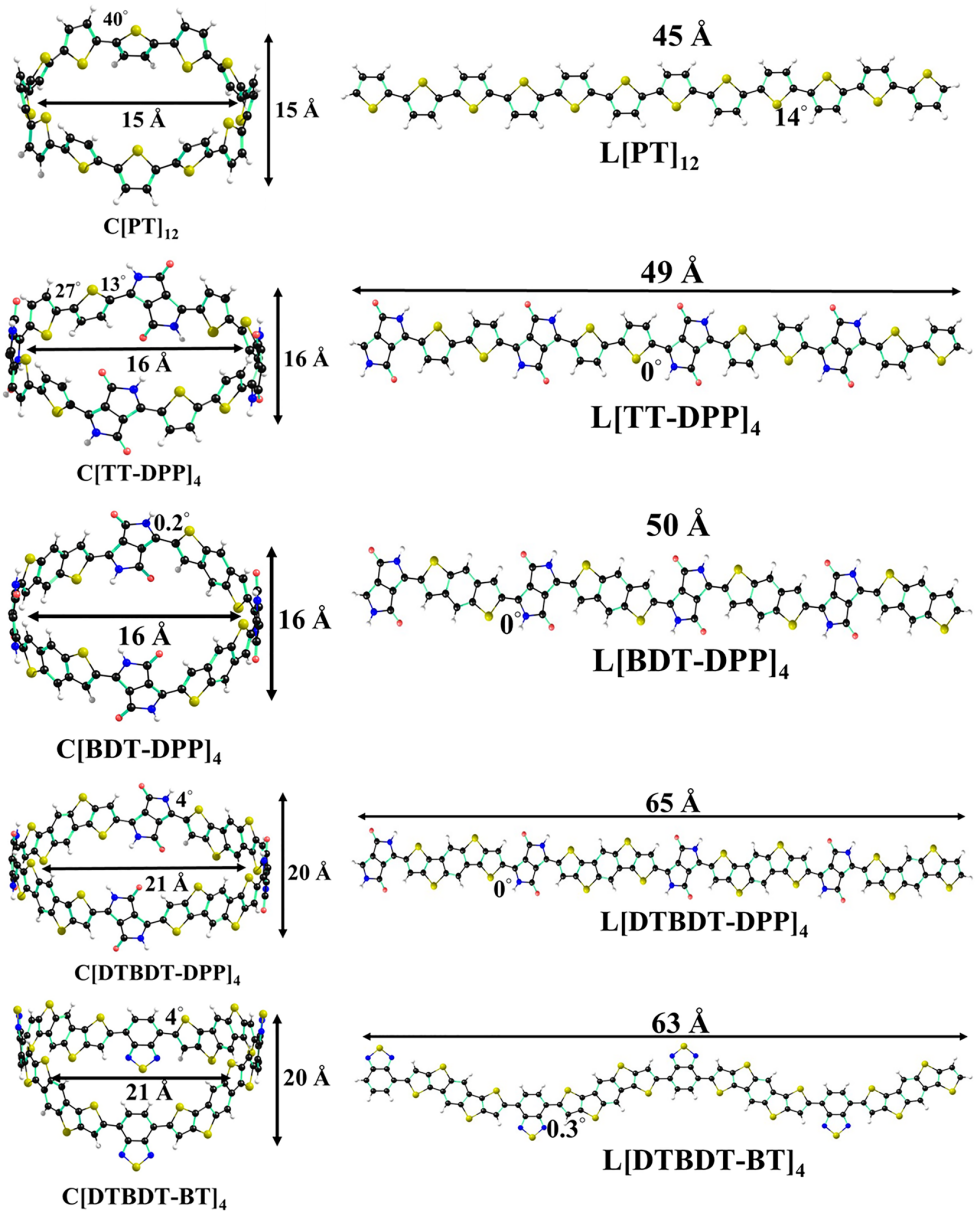
Optimized geometries of cyclic (C[PT]_12_, C[TT-DPP]_4_, C[BDT-DPP]_4_, C[DTBDT-DPP]_4_, and C[DTBDT-BT]_4_) and linear (L[PT]_12_, L[TT-DPP]_4_, L[BDT-DPP]_4_, L[DTBDT-DPP]_4_, and L[DTBDT-BT]_4_) donor–acceptor-based conjugated molecules as determined at B3LYP-D/6-31G(d,p) level of theory.

Optimized geometries of macrocyclic and linear compounds of [PT]_12_, [TT-DPP]_4_, [BDT-DPP]_4_, [DTBDT-DPP]_4_, and [DTBDT-BT]_4_ are shown in Fig. 2, along with important dihedral angles obtained at B3LYP-D/6-31G(d,p) level of theory. Optimized geometries of smaller-size cyclic and linear compounds are depicted in Figs. S1 and S2. It can be seen from Fig. 2, Figs. S1 and S2 that the cyclic structures (C[PT]_12_, C[TT-DPP]_4_, C[BDT-DPP]_4_, C[DTBDT-DPP]_4_, and C[DTBDT-BT]_4_), have twists in the dihedral angle (14°–18°) whereas linear oligomers (L[PT]_12_, L[TT-DPP]_4_, L[BDT-DPP]_4_, L[DTBDT-DPP]_4_, and L[DTBDT-BT]_4_) have a co-planar backbone. C[PT]_12_ exhibits larger dihedral angles between adjacent thiophenes among all macrocyclic compounds. The calculated dihedral angles between adjacent thiophenes range from 40° to 51° for C[PT]_8_, C[PT]_10_, and C[PT]_12_ models. Linear polythiophenes (L[PT]_8_, L[PT]_10_, and C[PT]_12_) have a planar structure with an end-to-end distance of 30 Å, 37 Å and 45 Å, respectively. On cyclization, C[PT]_8_, C[PT]_10_, and C[PT]_12_ have a circular belt shape with diameters of 8 Å, 12 Å and 15 Å, respectively. In the case of TT-DPP-based compounds, linear oligomers (L[TT-DPP]_2_, L[TT-DPP]_3_, and L[TT-DPP]_4_) have shown co-planar structures with the corresponding end-to-end distance of 24 Å, 36 Å and 49 Å, respectively. In the respective cyclic counterparts (C[TT-DPP]_2_, C[TT-DPP]_3_, and C[TT-DPP]_4_), the values of dihedral angle between two thiophene rings are 5°, 17° and 27°. The dihedral angles between thiophene and DPP units are 1°, 17° and 34°, respectively. Similar to thiophene-based macrocycles, the donor–acceptor-based conjugated C[TT-DPP)_n_ macrocycles also have the belt shape structure. The diameters of C[TT-DPP]_2_, C[TT-DPP]_3_, and C[TT-DPP]_4_ are 8 Å, 12 Å and 16 Å, respectively.

Unlike thiophene-based macrocycles, [BDT-DPP]_n_, [DTBDT-DPP]_n_, and [DTBDT-BT]_n_ have shown dihedral angle values close to 0°. Weak intermolecular interactions between oxygen atoms of DPP units and hydrogen atoms of BDT/DTBDT are responsible for smaller dihedral angles between the adjacent units. The absence of such interactions leads to twists in the dihedral angles in the cases of C[PT]_n_. C[BDT-DPP]_4_ has shown a circular shape with diameters of 16 Å; C[BDT-DPP]_3_ formed a reuleaux triangle shape with a diameter of 12 Å, whereas C[BDT-DPP]_2_ shows an oval shape with diameters of an 8 Å and 7 Å. Linear molecules of DTBDT-DPP (L[DTBDT-DPP]_2_, L[DTBDT-DPP]_3_, L[DTBDT-DPP]_4_) show end-to-end lengths 32 Å, 49 Å and 65 Å. The top view of all optimized conjugated macrocycles is depicted in Fig. S3. The C[DTBDT-DPP]_4_ macrocycle with four repeating units is arranged in a squircle (an intermediate shape between a square and a circle) shape with a diameter of 21 Å, whereas C[DTBDT-DPP]_3_ forms a reuleaux triangle shape with a diameter of 16 Å. The smaller macrocycle C[DTBDT-DPP]_2_ arranges in an oval shape (with a diameter of 12 Å and 10 Å). Due to the different morphology of the [DTBDT-BT]_n_ backbone (n = 2–4), the linear oligomers show a zigzag arrangement of donor and acceptor units with diameters of 31 Å, 47 Å and 63 Å. Four repeating units of macrocyclic DTBDT-BT ring display a crown shape with a diameter of 20 Å, whereas C[DTBDT-BT]_3_ arranged in a reuleaux triangle shape with a diameter of 15 Å, and C[DTBDT-BT]_2_ shows an oval shape with diameters of 12 Å and 9 Å.

### Strain energy

The energy associated with deforming a linear conjugated molecule when it is included in a conjugated macrocycle is defined as the macrocyclic strain energy (SE). SE in cyclic organic molecules arises due to the deviation in the structural parameters from their ideal angle to achieve maximum stability in a specific conformation. The SE arises due to the distortions of bond lengths and torsion angles from the typical values^32^. The energy associated with the formation of macrocyclic molecules from linear molecules is referred to as the macrocyclic strain energy^23,33^. The calculated strain energies for all the macrocyclic compounds using B3LYP-D/6-31G** are presented in Table 1. The calculated SE values of conjugated macrocycles are in the same range as the SE of previously reported macrocycles^16,32–35^.

**Table 1:** Calculated strain energies (in kcal/mol) of designed macrocyclic molecules determined at B3LYP-D/6-31G** level of theory.

Compound	Strain energy (kcal/mol)		
	n = 8	n = 10	n = 12
C[PT]_n_	59.41	46.54	37.98

	n = 2	n = 3	n = 4
C[TT-DPP]_n_	85.37	41.79	29.76
C[BDT-DPP]_n_	85.89	54.07	38.87
C[DTBDT-DPP]_n_	63.55	40.02	28.57
C[DTBDT- BT]_n_	80.07	53.68	40.71

In cases of small and medium rings (C[PT]_8_, C[PT]_10_, two and three repetitive units of TT-DPP, BDT-DPP, DTBDT-DPP, and DTBDT-BT) exhibit higher strain energy due to bending of the coplanar aromatic structures. Larger macrocyclic rings C[PT]_12_, C[TT-DPP]_4_, C[BDT-DPP]_4_, C[DTBDT-DPP]_4_, and C[DTBDT-BT]_4_ have less strain energies of 37.98, 29.76, 38.87, 28.57 and 40.71 kcal/mol, respectively. C[TT-DPP]_4_ and C[DTBDT-DPP]_4_ show less strain energy of 29.8 kcal/mol and 28.6 kcal/mol. The smaller strain energies in the case of C[TT-DPP]_4_ is attributed to the presence of more dihedral angles between the adjacent rings. Having more dihedral angles provided conformational flexibility during cyclization. In the cases of other macrocycles, especially the D–A-based C[BDT-DPP]_n_ and C[DTBDT-BT]_n_ have fewer numbers of dihedral angles and larger fused aromatic rings. As a result, more molecules have to undergo more strain when they cyclize. Again, the lack of weak intermolecular interactions between thiophene units leads to more strain when the linear thiophene chain cyclizes. Smaller strain energy is observed in the squircle-shaped C[DTBDT-DPP]_4_ ring (28.6 kcal/mol) compared to the crown-shaped C[DTBDT-BT]_4_ ring (40.71 kcal/mol).

### Radial π-conjugation

We have evaluated the highest occupied molecular orbital (HOMO) and the lowest unoccupied molecular orbital (LUMO) energies of C[TT-DPP]_4_ and L[TT-DPP]_4_ using three different functionals, viz., B3LYP, CAM-B3LYP, and mPW1PW91 functionals using the optimized geometries. The calculated results are compared with the previously reported experimental values (Table S1). We found that the mPW1PW91 functional can predict the HOMO and LUMO values comparable with the experimental values. Thus, we have considered the mPW1PW91 functional to evaluate the frontier energy values for both linear and cyclic molecules.

**Figure 3:**
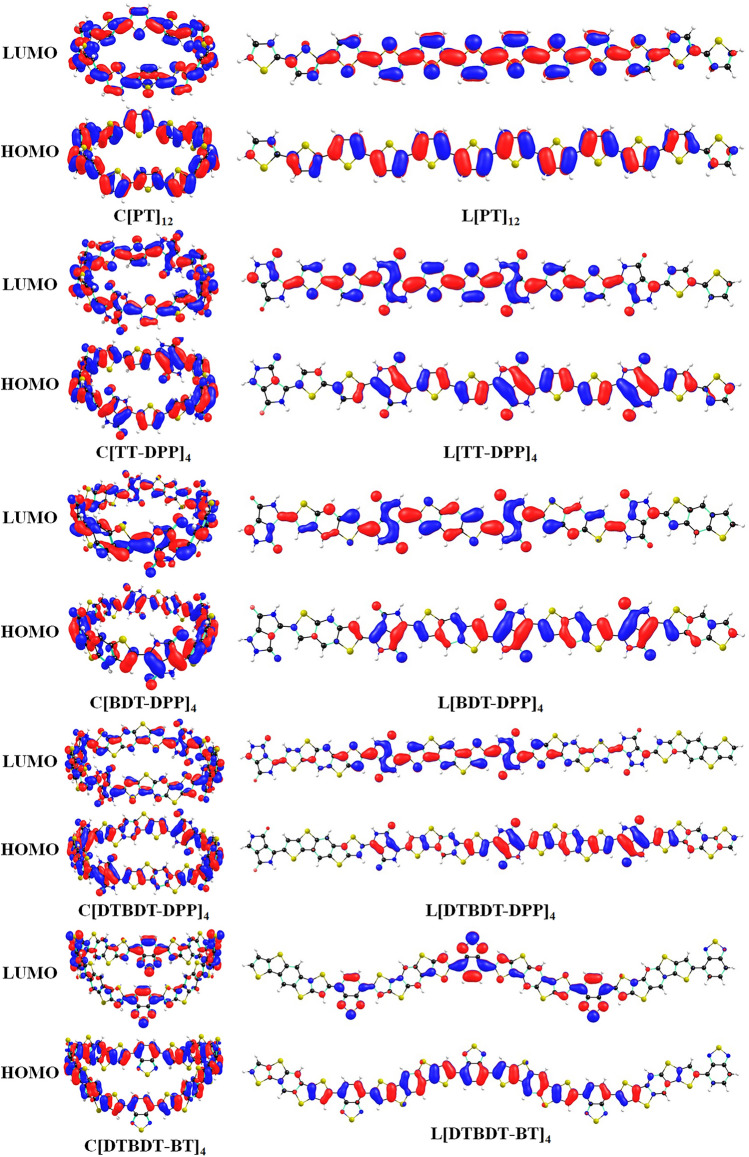
Pictorial representation of HOMO and LUMO wave functions of cyclic (C[PT]_12_, C[TT-DPP]_4_, C[BDT-DPP]_4_, C[DTBDT-DPP]_4,_ and C[DTBDT-BT]_4_) and linear (L[PT]_12_, L[TT-DPP]_4_, L[BDT-DPP]_4_, L[DTBDT-DPP]_4,_ and L[DTBDT-BT]_4_) molecules obtained at mPW1PW91/6-31G(d,p) level of theory.

HOMO, LUMO, and the difference between HOMO and LUMO energies (E_g_) are the important factors that influence the optoelectronic properties and charge carrier transport properties of π-conjugated materials^20,36,37^. The pictorial representation of HOMO and LUMO wavefunctions obtained at the mPW1PW91/6-31G** level of theory for cyclic (C[PT]_12_, C[TT-DPP]_4_, C[BDT-DPP]_4_, C[DTBDT-DPP]_4,_ and C[DTBDT-BT]_4_) and linear (L[PT]_12_, L[TT-DPP]_4_, L[BDT-DPP]_4_, L[DTBDT-DPP]_4,_ and L[DTBDT-BT]_4_) molecules are depicted in Fig. 3. One can identify the difference in the π-electron distribution in cyclic and linear molecules. The radial π-conjugation can be observed in the cases of conjugated macrocycles, whereas the linear molecules show perpendicular π-conjugation. Also, except in the cases of C[DTBDT-DPP]_4_ and C[DTBDT-BT]_4_, in all other cyclic molecules, HOMO and LUMO wavefunctions are delocalized entire ring. Such delocalization indicates the formation of infinite radial π-conjugation. The HOMO delocalized on the entire ring, whereas the LUMO predominantly localized on acceptor units of C[DTBDT-DPP]_4_ and C[DTBDT-BT]_4_ (Fig. 3). Overall, replacing thiophene units with the electron-rich and electron-poor units in the conjugated macrocycles leads to fascinating electronic properties. In the cases of linear molecules, HOMO and LUMO wavefunctions are localized on a few repeating units. This suggests that the conjugated macrocycles systems can offer unique electronic and optical properties due to infinite π-conjugation and shape-persistent cycle structure compared to the conventional linear π-conjugated oligomers.

**Figure 4:**
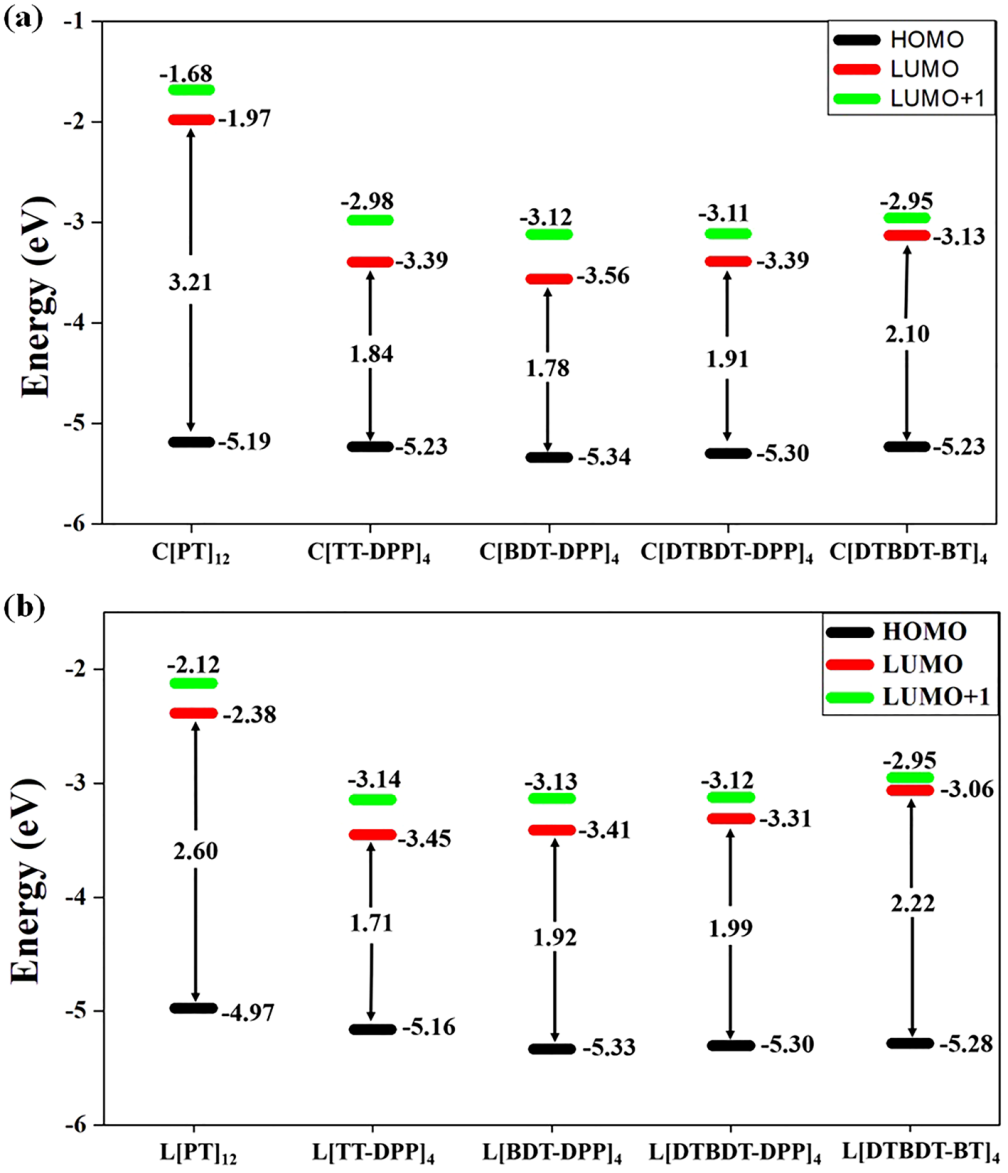
Calculated HOMO and LUMO energy levels of (**a**) cyclic (C[PT]_12_, C[TT-DPP]_4_, C[BDT-DPP]_4_, C[DTBDT-DPP]_4,_ and C[DTBDT-BT]_4_) and (**b**) linear (L[PT]_12_, L[TT-DPP]_4_, L[BDT-DPP]_4_, L[DTBDT-DPP]_4,_ and L[DTBDT-BT]_4_) donor–acceptor-based conjugated molecules determined at mPW1PW91/6-31G(d,p) level of theory. All values are in eV.

The calculated energies of HOMO–LUMO gaps and the energy difference between LUMO and LUMO + 1 for the larger systems are depicted in Fig. 4. The same energy values for all molecules considered in this study are reported in Table S2. Out of five systems considered in this study, we observed different electronic properties in the cases of PT and TT-DPP systems. We noted that HOMO energy levels are stabilized, and LUMO energy levels are destabilized when linear L[PT]_12_ and L[TT-DPP]_4_ molecules are cyclized (Fig. 4). In the [BDT-DPP]_4_, [DTBDT-DPP]_4_, and [DTBDT-BT]_4_, both HOMO and LUMO wavefunctions are slightly stabilized when the linear one cyclizes.

Similarly, significant shrinkage in the HOMO–LUMO gaps is observed in the cases of C[PT]_n_, C[TT-DPP]_n_ systems when the size of the ring is increased. However, marginal or no changes in the HOMO–LUMO gaps are noted in the other systems. This reveals the intriguing property of radial π-conjugation. As both PT and TT-DPP-based conjugated rings exhibit more radial π-conjugation character, the electronic properties of these molecules are different from other cases. Also, as expected, variation in the HOMO, LUMO, and HOMO–LUMO gaps values are observed depending on the electron-donating and the electron-accepting nature of donor and acceptor units. Overall, the choice of donor and acceptor units impacts the radial π-conjugation character along with the energy level alteration. Also, a good correlation between the energy gap between LUMO and LUMO + 1 (ΔE_(LUMO+1)-LUMO_) of non-fullerene acceptors and power conversion efficiency (PCEs) is shown^37,38^. From Table S2, it is observed that in both linear and cyclic molecules designed, larger size compounds have the lower ΔE_(LUMO+1)-LUMO_ when compared to smaller size compounds. Moreover, the ΔE_(LUMO+1)-LUMO_ gap of larger cyclic compounds has similar values as linear compounds.

### Reorganization energies

As described in the previous sections, the conjugated macrocycles offer unique electronic properties compared to linear counterparts due to the orientation of π-orbitals. Recent studies highlighted that the radially π-conjugated materials based on conjugated macrocycles could offer a much larger conductance modulation range than linear oligomers^39^. Thus, it is important to explore the charge transport properties of conjugated macrocycles. The internal reorganization energy (λ) is one of the key determinants of charge transport in organic materials^37,40–42^. The charge mobility exhibits an inverse relationship with reorganization energies, i.e., the lesser the λ value faster the charge mobility^20^. The calculated electron (λ_−_) and hole (λ_+_) reorganization energies for all the cyclic and linear compounds are listed in Table 2. The calculated hole and electron reorganization energy values for cyclic rings range from 0.09 to 0.26 eV and from 0.06 to 0.32 eV, respectively. The same values in linear oligomers range from 0.09 to 0.20 eV and from 0.06 to 0.19 eV, respectively. Slightly higher reorganization energies are observed in the cyclic structures than in the linear oligomers.

**Table 2:** Calculated energies (in eV) of hole (λ_+_) and electron (λ_-_) reorganization of cyclic and linear compounds (eV) calculated at B3LYP-D/6-31G** level of theory.

Compound	Linear		Cyclic	
	λ_+_	λ_−_	λ_+_	λ_−_
[PT]_8_	0.12	0.26	0.49	0.54
[TT-DPP]_2_	0.23	0.20	0.36	0.40
[BDT-DPP]_2_	0.20	0.18	0.28	0.21
[DTBDT-DPP]_2_	0.09	0.07	0.21	0.16
[DTBDT-BT]_2_	0.17	0.12	0.20	0.13
[PT]_10_	0.23	0.22	0.35	0.41
[TT-DPP]_3_	0.20	0.18	0.23	0.29
[BDT-DPP]_3_	0.08	0.07	0.18	0.14
[DTBDT-DPP]_3_	0.07	0.05	0.14	0.10
[DTBDT-BT]_3_	0.12	0.09	0.13	0.09
[PT]_12_	0.20	0.19	0.26	0.32
[TT-DPP]_4_	0.12	0.11	0.17	0.21
[BDT-DPP]_4_	0.10	0.08	0.13	0.11
[DTBDT-DPP]_4_	0.16	0.15	0.10	0.07
[DTBDT-BT]_4_	0.09	0.06	0.09	0.06

In the case of cyclic polythiophenes, λ_+_ values are less than λ_−_ values. This indicates that the energy required for hole transfer is lower than the electron transfer process. It is observed that λ_−_ and λ_+_ values for cyclic compounds are in good correlation with the size of the ring. Smaller λ_+_ and λ_−_ values are observed in the larger rings and longer oligomers (Table 2). Reduction in λ_+_ and λ_−_ values are found when one of the thiophene units is replaced with the DPP unit in both linear and cyclic compounds. Since the difference between λ_+_ and λ_−_ values are marginal, cyclic/linear [PT]_n_ and [TT-DPP]_n_ can be used as ambipolar molecules. Replacement of bithiophene units (TT) with BDT (C[BDT-DPP]_2_ to C[BDT-DPP]_4_ and L[BDT-DPP]_2_ to L[BDT-DPP]_4_) leads to a reduction in λ_+_ and λ_−_ values compared to linear and cyclic TT-DPP-based molecules. Furthermore, smaller λ_−_ values were noted than the λ_+_ energies. A small increment in the λ_+_ and λ_−_ values are observed when BDT unit replaced with DTBDT units (C[DTBDT-DPP]_2_ to C[DTBDT-DPP]_4_ and L[DTBDT-DPP]_2_ to L[DTBDT-DPP]_4_). Here also smaller λ_−_ values are noted than the λ_+_ values. Further modification of DPP unit with BT (C[DTBDT-BT]_2_ to C[DTBDT-BT]_4_ and L[DTBDT-BT]_2_ to L[DTBDT-BT]_4_) results decreased λ_+_ and λ_−_ values.

### Open-circuit voltage

The open-circuit voltage (V*oc*) is an important factor in considering the device performance of any photovoltaic material and its operating mechanism. V*oc* can be explained as the entire quantity of current provided by the photovoltaic device without any external load for electricity generation^43,44^. The V*oc* is strongly related to fill factor and power conversion efficiency^45^. The V*oc* can be calculated by the following Eq.^46–48^.

1 $$V_{OC} = \frac{1}{{\text{e}}}(E_{HOMO} \left( D \right) - E_{LUMO} \left( A \right)) - 0.3$$

Here, 0.3 is the voltage drop parameter, signifying that the open-circuit voltage can be more or less than 0.3, and e represents the element electron.

**Figure 5:**
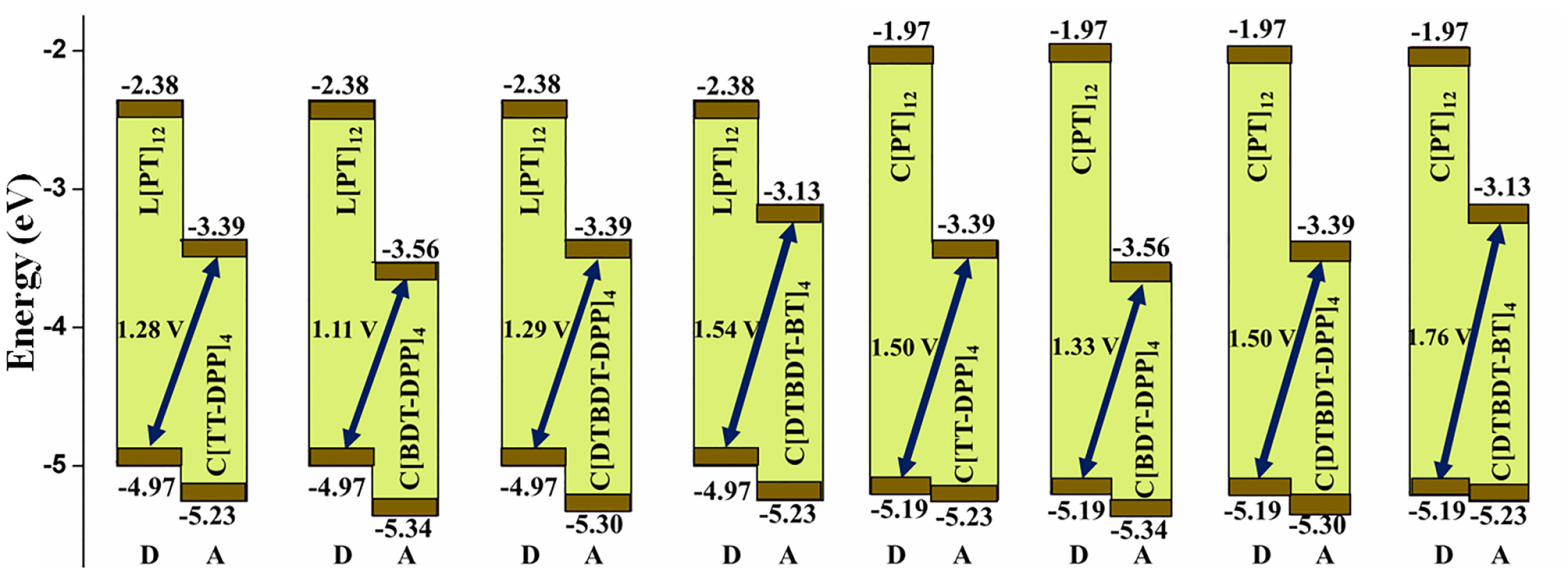
Open circuit voltage (V*oc*) of larger macrocyclic compounds with L[PT]_12_ and C[PT]_12_ as donor component.

Usually, for successful exciton dissociation at the D–A interface and efficient CT acquisition from donor to acceptor, the energetic driving force (L_D_-L_A_), defined as the difference in LUMO energy between the donor and acceptor, should be more than 0.3 eV. By considering this criterion, calculated the V*oc* for designed macrocyclic compounds by considering linear and cyclic compounds as donor materials. The outcome of these parameters is presented in Figs. 5 and S4 and Table S3. All larger macrocyclic compounds have shown the acceptable V*oc* with L[PT]_12_ and C[PT]_12_ as donor materials. Among all combinations, C[PT]_12_ as the donor and C[DTBDT-BT]_4_ as the acceptor, and L[PT]_12_ as the donor and C[DTBDT-BT]_4_ as the acceptor, exhibit larger Voc of 1.76 V and 1.54 V respectively due to lower HOMO energy of C[PT]_12_ and L[PT]_12_ and higher LUMO of C[DTBDT-BT]_4_. Two combinations, L[DTBDT-BT]_4_ as the donor and C[DTBDT-DPP]_4_ as the acceptor, L[DTBDT-BT]_4_ as the donor and C[TT-DPP]_4_ as the acceptor, have also shown a high V_oc_ of 1.6 V and 1.59 V. Based on these results, it is predicted that C[DTBDT-BT]_4_ could be a promising acceptor material to enhance power conversion efficiency by virtue of better charge conduction properties.

### Excited-state analysis

Time-dependent DFT (TD-DFT) calculations were carried out at the TD-mPW1PW91/6-31G(d,p) level of theory to elucidate the absorption properties of these molecules. The calculated optical properties of the five lowest excited states in cyclic rings (S_1_–S_5_) and the lowest excited state energies (S_1_) of linear oligomers with their oscillator strength (f) are shown in Tables 3 and S4. In the cases of C[PT]_12_, C[TT-DPP]_4_, and L[TT-DPP]_4_, the calculated absorption energies are in good agreement with the experimental reports^6,49^.

**Table 3:** Calculated excited state energies (in eV) and oscillator strengths (au) of cyclic and linear molecules determined at mPW1PW91/6-31G** level of theory.

	Cyclic				Linear	
	S_1_	f	S_2_	f	S_1_	f
[PT]_8_	3.08	0.64	3.67	0.64	2.33	2.78
[TT-DPP]_2_	0.78	0.77	1.94	0.09	1.88	1.91
[BDT-DPP]_2_	1.19	0.39	2.15	0.02	2.00	1.69
[DTBDT-DPP]_2_	1.40	0.89	2.15	0.00	1.95	2.53
[DTBDT-BT]_2_	1.41	0.36	1.97	0.23	1.99	1.54
[PT]_10_	2.78	1.26	3.32	1.26	2.19	3.52
[TT-DPP]_3_	1.49	1.10	2.16	0.01	1.58	3.09
[BDT-DPP]_3_	1.27	1.04	1.99	0.04	1.84	2.94
[DTBDT-DPP]_3_	1.46	1.75	2.02	0.04	1.74	3.99
[DTBDT-BT]_3_	1.54	0.60	1.97	0.59	1.84	2.94
[PT]_12_	2.58	1.82	3.04	1.82	2.11	4.25
[TT-DPP]_4_	1.35	1.90	1.90	0.02	1.43	4.24
[BDT-DPP]_4_	1.31	1.74	1.86	0.10	1.60	3.94
[DTBDT-DPP]_4_	1.49	1.67	1.92	1.08	1.65	5.52
[DTBDT-BT]_4_	1.62	1.60	1.93	1.60	1.78	4.15

As expected, shrinkage in the optical gaps is observed as the length of the oligomer increases. The calculated optical gaps in the L[PT]_12_ and L[TT-DPP]_4_ are 2.11 and 1.43 eV, respectively. The lowest absorption value is substantially red-shifted ~ 300 nm when thiophene units are replaced with the DPP units. The introduction of DPP units stabilized the LUMO in L[TT-DPP]_n_ oligomers. As a result, smaller optical gaps were observed in the DPP-based oligomers. Again, slight blue shifts in absorption values are observed when TT units are replaced with the electron-rich BDT/DTBDT units.

**Figure 6:**
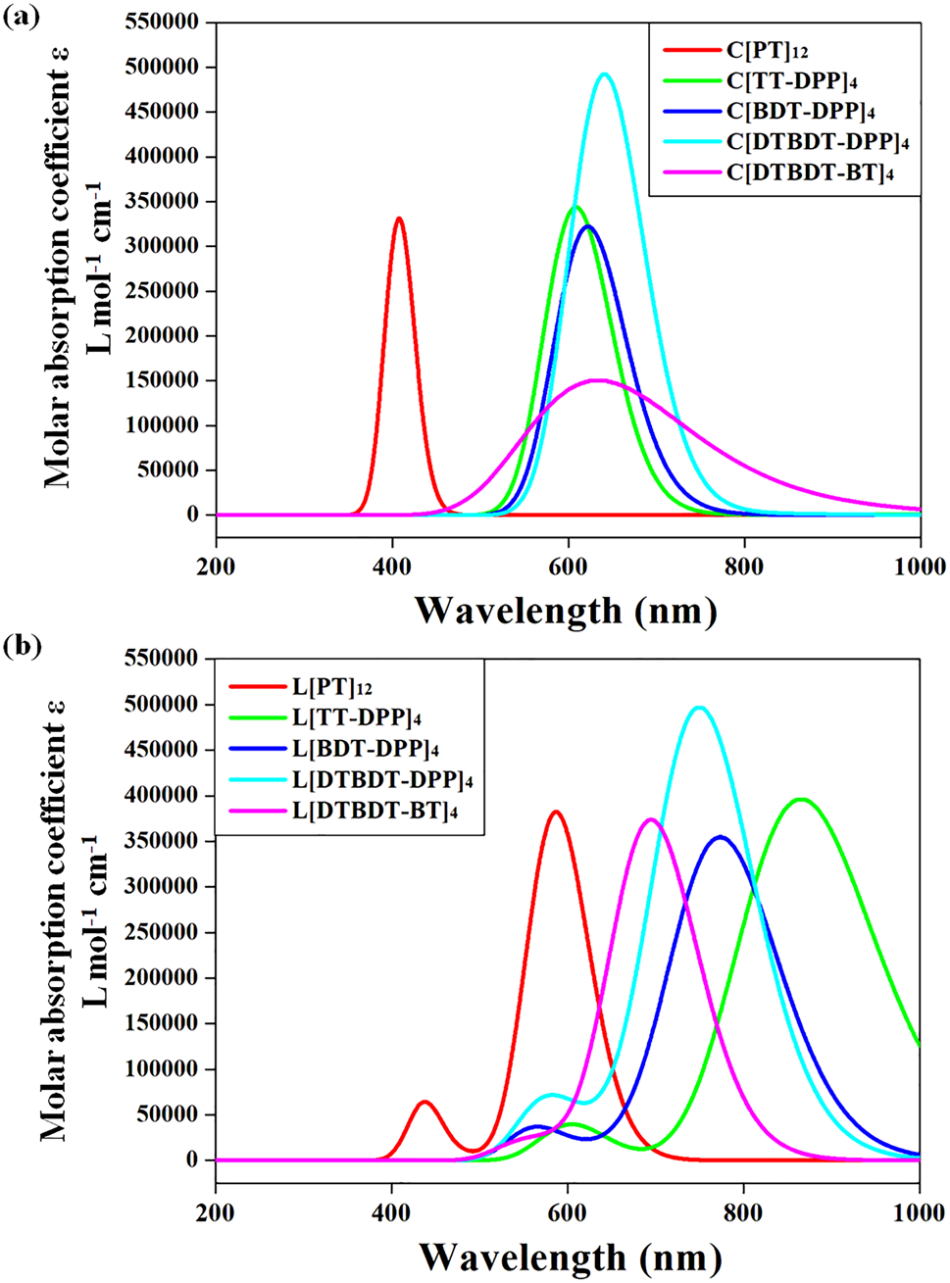
Graphical representation of absorption wavelengths of larger (**a**) linear and (**b**) macrocyclic compounds calculated at mPW1PW91/6-31G(d,p) level of theory.

Strong blue-shift in the absorption energies are noted in the cyclic rings compared with the linear oligomers. The graphical representation of absorption wavelengths of larger linear and macrocyclic compounds is shown in Fig. 6. Macrocyclic compounds of TT-DPP, BDT-DPP, DTBDT-DPP, and DTBDT-BT show absorption at a longer wavelength (redshift) than their linear counterparts. The oscillator strength of macrocyclic compounds is zero by cyclization of linear compounds. One of the primary differences between cyclic and linear geometries is the degrees of freedom. The closed-loop formation by joining both termini reduces the degrees of freedom and conformational disorder. The lowest excited state excitation is forbidden for cyclic structures^50^. Thus, the lowest excitations in cyclic compounds originate from the S_0_ ≥ S_2_ transitions.

**Figure 7:**
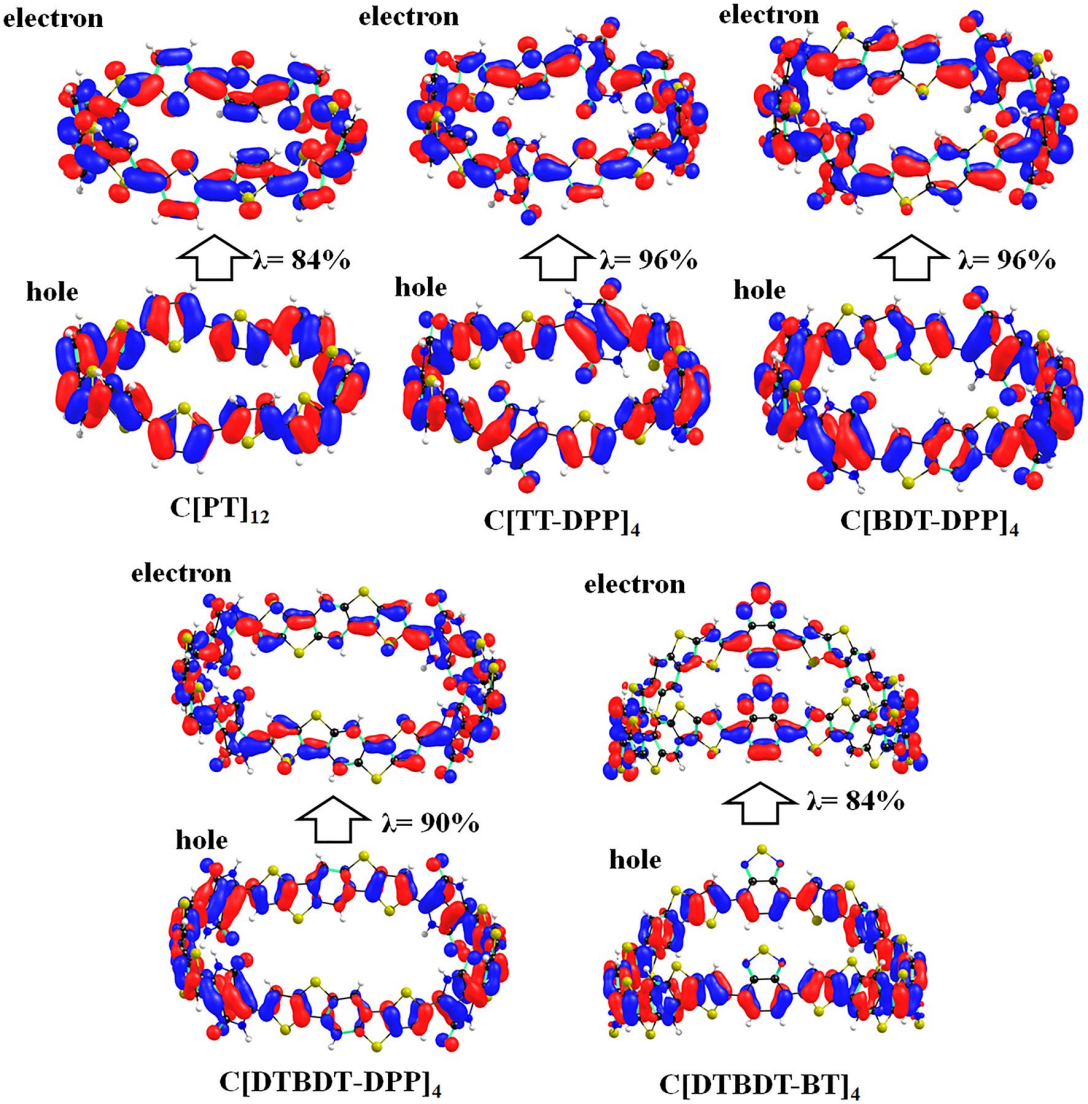
Pictorial representation of the natural transition orbitals of linear (C[PT]_12_, C[TT-DPP]_4_, C[BDT-DPP]_4_, C[DTBDT-DPP]_4_, and C[DTBDT-BT]_4_) molecules corresponds to their lowest excited state (S_1_) calculated at TD-mPW1PW91/6-31G(d,p) level of theory. Where, λ is the fraction of the hole–electron contribution to the excitation.

**Figure 8:**
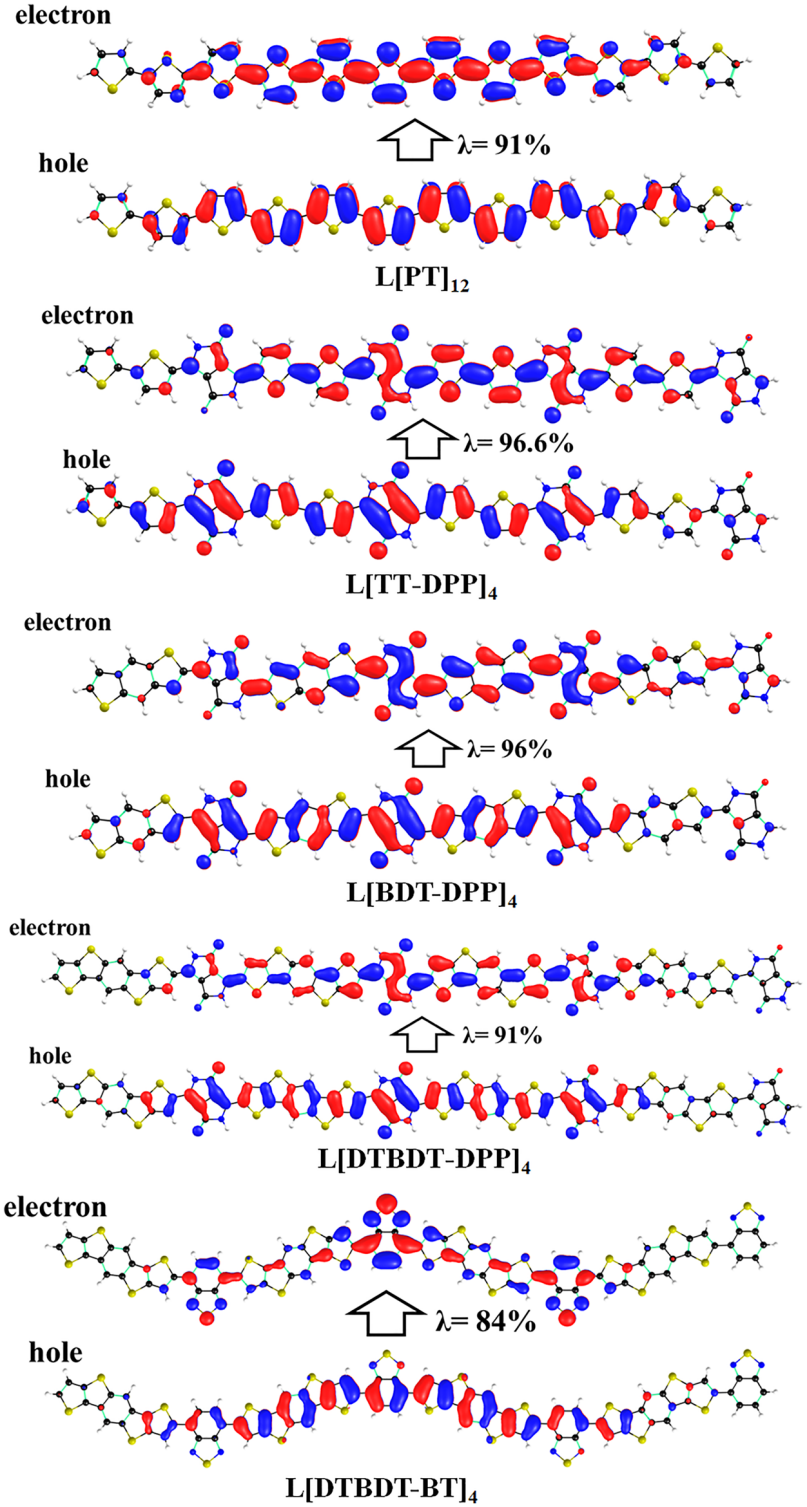
Pictorial representation of the natural transition orbitals of linear (L[PT]_12_, L[TT-DPP]_4_, L[BDT-DPP]_4_, L[DTBDT-DPP]_4_, and L[DTBDT-BT]_4_) molecules corresponds to their lowest excited states (S_1_) calculated at TD-mPW1PW91/6-31G(d,p) level of theory. Where, λ is the fraction of the hole–electron contribution to the excitation.

Further, NTO analysis was carried out to characterize the nature of the excited state. The calculated hole and electron wavefunctions of larger cyclic and linear molecules are depicted in Figs. 7 and 8. The same for smaller oligomers is presented in Figs. S5–S8. The hole and electrons are delocalized across the backbone in the linear oligomers. Substantial overlap between hole and electron wavefunctions is observed in the lowest excited states of all oligomers. Thus, one can confirm that these states are Frenkel-type excitations (electron–hole pairs are localized at the single molecular unit). Strong oscillator strengths are also noted in all cases.

The lower excitation transitions are forbidden for conjugated macrocyclic compounds, unlike linear oligomers. Thus, we have evaluated the lowest five excited states using the TD-DFT method (Table S4). From these calculations, we found the bright oscillator strengths in the second-excited state S_2_ in the cases of C[PT]_12_, C[DTBDT-DPP]_4_, and C[DTBDT-BT]_4_. S_0_ ≥ S_4_ transition is predicted as a bright state in the cases of C[TT-DPP]_4_ and C[BDT-DPP]_4_. We have calculated the NTOs for these excitations to understand the nature of the excited state (Fig. S9). From Fig. S9, the NTO analysis on the S_2_ state of C[PT]_12_ and C[DTBDT-DPP]_4_; and the S_4_ state in C[TT-DPP]_4_ and C[BDT-DPP]_4_ indicate that the hole and electron wavefunctions are localized throughout the compound which refers Frenkel-type excitations. In C[DTBDT-BT]_4_, the second excited state exhibits hybridized local and charge-transfer (HLCT) state. Here, the hole wavefunction is delocalized on entire molecule and electron wavefunction is dominantly localized on two opposite BT acceptor units.”

### Exciton binding energy and singlet–triplet gap

The singlet–triplet energy gap (ΔE_ST_), which is the energy difference between the lowest non-charge transfer singlet (S_1_) and triplet (T_1_) excited states, is an important parameter for OSC material. The exciton dissociation process occurs through singlet-CT states. Nongeminate or bimolecular recombination may occur during charge migration; this results in the creation of CT excitons, which can be of singlet or triplet character. Through back electron transfer triplet-CT, the state relaxes to the T_1_ state, and recombination occurs. Due to the large difference between CT state energy and T_1_ energy, the speed of T_1_ to thermalize back into triplet-CT will be limited if the CT driving force is small (as required to maximize open-circuit voltage, V_*OC*_). In order to reduce both voltage loss and nongeminate recombination, the ΔE_ST_ needs to be minimized^51,52^. The calculated ΔE_ST_ values for larger macrocyclic and linear compounds are included in Table 4. Indeed, from Table 4, the energies of S_1_ and T_1_ of macrocyclic compounds are lower than corresponding linear compounds, thus reducing the ΔE_ST_ in macrocyclic compounds (~ 0.41 to 0.49 eV) compared to linear (~ 0.46 to 0.67 eV). Among macrocyclic compounds C[TT-DPP]_4_ and [DTBDT-BT]_4_ consists of lower ΔE_ST_ of 0.41 eV and 0.43 eV. As a result, it appears that cyclization of linear compounds can effectively minimize voltage loss and nongeminate recombination by lowering the ΔE_ST_.

**Table 4:** Calculated lowest singlet (S_1_), triplet (T_1_) excitation energies, Singlet–triplet gap ΔE_ST_ and exciton binding energy E_b_ of larger macrocyclic and linear compounds in eV obtained at mPW1PW91/6-31G** level of theory.

	Cyclic				Linear			
	S_1_	E_b_	T_1_	ΔE_ST_	S_1_	E_b_	T_1_	ΔE_ST_
[PT]_12_	2.58	0.63	2.09	0.49	2.11	0.48	1.52	0.59
[TT-DPP]_4_	1.35	0.49	0.94	0.41	1.43	0.28	0.81	0.62
[BDT-DPP]_4_	1.31	0.47	0.88	0.44	1.60	0.32	0.93	0.67
[DTBDT-DPP]_4_	1.49	0.42	1.00	0.48	1.65	0.33	1.01	0.64
[DTBDT-BT]_4_	1.62	0.48	1.19	0.43	1.78	0.44	1.33	0.46

Further, the exciton separation procedure leads to additional energy losses because of the high exciton binding energy E_b_. Large exciton binding energy must be overcome to dissociate exciton to charges successfully^44,46,53^. To overcome this, one of the key parameters is E_b_, which is directly related to the charge separation in OSCs. It can be calculated theoretically using following expression^44,52,54^.

2 $${\text{E}}_{{\text{b}}} = {\text{IP}} - {\text{EA}} - {\text{E}}_{{{\text{opt}}}}$$

Where IP and EA are the ionization potential and electron affinity, respectively; and E_opt_ is the optical band gap.

The E_b_ of larger macrocyclic and linear compounds were calculated and given in Table 4. The table showed that macrocyclic compounds showed little more E_b_ values of difference ~ 0.14 to 0.2 eV when compared to linear compounds. Among the designed macrocyclic compounds, C[DTBDT-DPP]4, C[BDT-DPP], and C[DTBDT-BT]4 possess the lower E_b_.

### Electronic coupling

The charge transfer properties of organic molecules are primarily determined by lower reorganization energy and electronic coupling between molecules. The HOMO–HOMO coupling accelerates hole transport, whereas the LUMO–LUMO coupling enhances the electron transport. We evaluated the electronic couplings of dimer configurations of macrocyclic compounds in three packing arrangements. The first packing mode is the interaction of an acceptor unit of one macrocyclic ring with the acceptor unit of another ring (AA). The second packing mode corresponds to the acceptor unit of one ring interacting with the donor unit of another ring (AD). And the third packing mode is the donor unit of one ring with the donor unit of another ring (DD) (Fig. S10). The distance between the rings is fixed at 3.5 Å in all complexes. All transfer integral values are calculated using the B3LYP/6-31G(d,p) level of theory. All the findings of electronic coupling values are tabulated in Table 5. In the case of C[PT]_12_, we found similar HOMO–HOMO and LUMO–LUMO electronic coupling values. Also, we note lower reorganization energies (λ_+_ and λ_−_) are observed (Tables 2 and 5). Therefore C[PT]_12_ can be categorized as an ambipolar molecule. Modifying the thiophene unit with the DPP acceptor unit (C[TT-DPP]_4_) enhanced the LUMO–LUMO coupling in DD interacting configuration, whereas weaker coupling was observed in the DA configuration. In the case of C[BDT-DPP]_4_, substantial HOMO–HOMO coupling was seen in the AA configuration, whereas weak coupling was observed in the DA configuration. In the instance of [DTBDT-DPP]_4_, it has shown similar HOMO–HOMO and LUMO–LUMO coupling in all (AA, DA, and DD) configurations. Based on electronic coupling values and reorganization energies, one can confirm the ambipolar behavior of [DTBDT-DPP]_4_. When it comes to [DTBDT-BT]_4_, the electronic coupling values depend on the packing between the rings. More significant LUMO–LUMO coupling was observed in the AA configuration, and weaker LUMO–LUMO coupling was observed in the DD configuration. C[TT-DPP]_4_ and [DTBDT-BT]_4_ can act as electron acceptor materials based on strong LUMO–LUMO couplings and low electron reorganization energies. However, the molecular packing of macrocyclic molecules strongly influences the electronic coupling properties.

**Table 5:** Calculated transfer integral values (in meV) between conjugated macrocycles at B3LYP/6-31G** level of theory.

Compound	HOMO–HOMO coupling (meV)	LUMO–LUMO coupling (meV)
C[PT]_12_	16.96	16.68
**C[TT-DPP]**_**4**_		
AA	17.92	18.31
DA	1.01	0.87
DD	18.49	28.38
**C[BDT-DPP]**_**4**_		
AA	27.81	13.32
DA	1.73	3.85
DD	6.13	12.36
**C[DTBDT-DPP]**_**4**_		
AA	18.34	16.16
DA	13.84	16.30
DD	17.78	17.60
**C[DTBDT-BT]**_**4**_		
AA	7.63	31.20
DA	19.04	12.32
DD	13.88	3.74

### The hole/electron mobility

The intermolecular packing configurations of adjacent molecular segments have a considerable impact on the transfer integral. It has been widely reported that face-to-face parallel-stacking has greater orbital overlapping, resulting in a large-scale contribution to charge transfer in organic systems. To estimate the charge transport rate constants (*k*_*h*_ and *k*_*e*_) and mobilities (μ_h_ and μ_e_), we have considered in three packing configurations (Fig. S10) as described above in electronic coupling calculations. Hole and electron transport rate constant calculated using Eq. (6) and hole and electron mobilities calculated using Eq. (7) for different configuration of packings of larger macrocyclic compounds are tabulated in Table 6. From this table one can observe that the mode of packing configuration impacted the hole and electron rate constants and mobilities. Increasing the conjugation length in macrocyclic compounds increases the rate of mobilities of hole and electron. Among the macrocyclic compounds C[DTBDT-BT]_4_ showing the highest *k*_*e*_ and μ_e_ of 3.68 × 10^13^ S^−1^ and 0.88 cm^2^ V^−1^ S^−1^ due to highest LUMO–LUMO coupling and lower electron reorganization energy in AA configuration. As well in DA configuration it is showing hole transport rate of 8.42 × 10^12^ S^−1^ and hole mobility 0.2 cm^2^ V^−1^ S^−1^. Among the macrocyclic compounds C[BDT-DPP]_4_ consists of high hole transport rate 1.03 × 10^13^ S^−1^ and hole mobility 0.25 cm^2^ V^−1^ S^−1^.

**Table 6:** Calculated hole and electron charge transport rate constant *k*_*h*_*/k*_*e*_ (S^−1^), hole and electron mobility μ_h_/μ_e_ (cm^2^ V^−1^ S^−1^) using coupling values obtained from three different packing configurations of macrocyclic compounds with charge transport distance *r* (Å) between two macrocycles, obtained with Eq. (6) and (7).

	r (Å)	*k*_*h*_	μ_h_	*k*_*e*_	μ_e_
C[PT]_12_	3.5	1.55 × 10^12^	0.04	3.82 × 10^11^	0.01
**C[TT-DPP]**_**4**_					
AA	3.5	2.53 × 10^12^	0.06	1.54 × 10^12^	0.04
DA	3.5	7.80 × 10^9^	0.00	4.75 × 10^9^	0.00
DD	3.5	2.53 × 10^12^	0.06	3.73 × 10^12^	0.09
**C[BDT-DPP]**_**4**_					
AA	3.5	1.03 × 10^13^	0.25	2.94 × 10^12^	0.07
DA	3.5	5.26 × 10^10^	0.00	2.78 × 10^11^	0.01
DD	3.5	4.74 × 10^11^	0.01	2.50 × 10^12^	0.06
**C[DTBDT-DPP]**_**4**_					
AA	3.5	6.51 × 10^12^	0.16	8.23 × 10^12^	0.20
DA	3.5	3.94 × 10^12^	0.09	8.23 × 10^12^	0.20
DD	3.5	6.51 × 10^12^	0.16	1.04 × 10^13^	0.25
**C[DTBDT-BT]**_**4**_					
AA	3.5	1.49 × 10^12^	0.04	3.68 × 10^13^	0.88
DA	3.5	8.42 × 10^12^	0.20	5.51 × 10^12^	0.13
DD	3.5	4.57 × 10^12^	0.11	6.12 × 10^11^	0.01

### Properties of electron-donor and electron-acceptor blends

**Figure 9:**
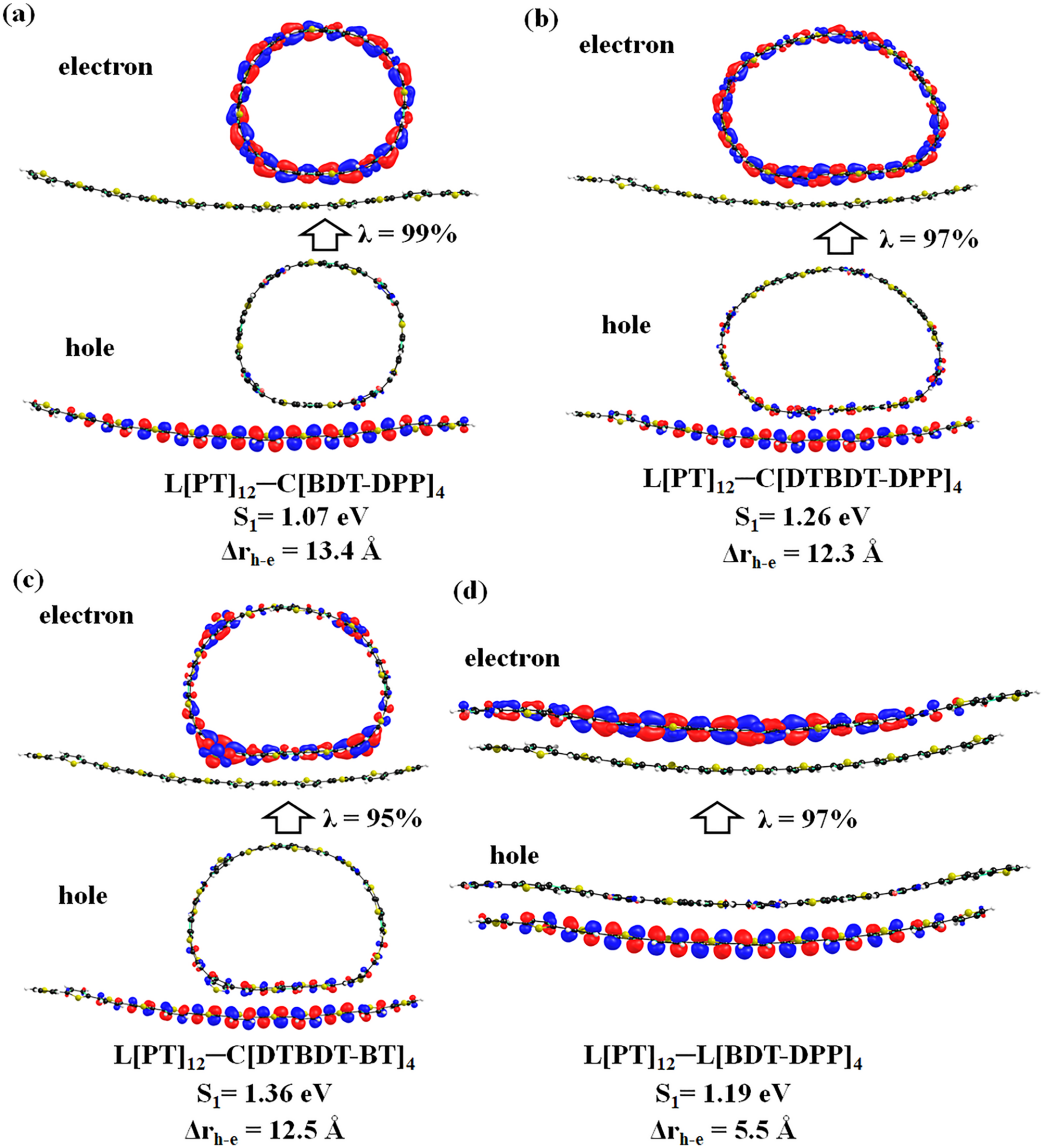
Pictorial representation of the charge transfer states of various cyclic-linear complexes (**a**) L[PT]_12_─C[BDT-DPP]_4_, (**b**) L[PT]_12_─C[DTBDT-DPP]_4_, (**c**) L[PT]_12_─C[DTBDT-BT]_4_ and linear–linear complex **(d)** L[PT]_12_─L[BDT-DPP]_4 _calculated at TD-B3LYP-D/6-31G(d,p) level of theory where λ is the fraction of the hole–electron contribution to the excitation.

It is clear from the discussion in previous sections that the conjugated D–A macrocycles can indeed be used as electron acceptor materials (similar to fullerenes and fullerene derivatives) for organic solar cell applications. We have constructed a model interface between electron donor (L[PT]_12_) and electron acceptor materials. The L[PT]_12_ and conjugated D–A macrocycles complexes are considered to gain insights into molecular packing at the interface, charge transfer states, and charge separation behavior. C[TT-DPP]_4_, C[BDT-DPP]_4_, C[DTBDT-DPP]_4_, and C[DTBDT-BT]_4_ have been selected as acceptor materials and L[PT]_12_ selected as donor material to model linear-cyclic interfaces. Further, these results are compared with the complexes which are made from linear oligomers. L[BDT-DPP]_4_ and L[PT]_12_ are considered as electron-acceptor and electron-donor materials, respectively. All the complexes were optimized at the B3LYP-D/6-31G(d,p) level of theory. Further, the excited state analysis was carried out on the ground-state optimized geometries. NTO analysis was also performed to understand the nature of excitation transition. One of the drawbacks of OSCs is that exciton lifetimes are typically very short due to the involvement of coulombically bound electron–hole pairs, resulting in short exciton diffusion lengths. Thus, an adequate driving force for charge separation is required for complete electron and hole separation. The spatial distance between the centroid of the hole and electron (Δr_h-e_), which is the key factor in charge separation, is obtained by the Multiwfn code^55^. Pictorial representation of charge-transfer states of all complexes and CT state energies and Δr_h-e_ values are shown in Fig. 9. Larger Δr_h-e_ values are observed in the complexes where linear electron-donor and cyclic electron-acceptor is considered. The electron wavefunction is delocalized away due to the cyclic structure of the acceptor molecule. As a result, a more significant Δr_h-e_ distance (~ 12–13 Å) in linear-acceptor complexes is observed. Among all interfaces, L[PT]_12_─C[BDT-DPP]_4_ showed the highest Δr_h-e_ of 13.4 Å. The DTBDT unit containing macrocyclic compounds exhibited more structural distortion when interacting with donor material. Hence less Δr_h-e_ when compared to C[BDT-DPP]_4_. In the case of linear electron-donor and linear electron-acceptor complexes, due to one-on-one packing, strong Coulomb interactions lead to a smaller Δr_h-e_ of 5.5 Å.

The calculated hole and electron charge transfer rates and rate of charge recombination (using Eq. (9)) of modeled interfaces are given in Table 7, along with free energy changes ΔG_CT_ and ΔG_CR_. The negative ΔG_CT_ and ΔG_CR_ values demonstrate that charge transfer and charge recombination are thermodynamically favourable processes. An ideal electron-donor and electron-acceptor interface should have a higher value of *k*_CT_ and lower *k*_CR_ to enable effective exciton dissociation and reduced charge recombination at the interface. To emphasize this the ratio of *k*_CT_/*k*_CR_ are also calculated. The ratio of hole *k*_CT_/*k*_CR_ followed the order C[DTBDT-BT]_4_ > C[DTBDT-DPP]_4_ > C[TT-DPP]_4_ > C[BDT-DPP]_4_. The same order is also observed in the case of the ratio of electron *k*_CT_/*k*_CR._ As per Marcus’ theory, reorganization energy and electronic coupling are the two factors which influence the charge transfer rate. Strong LUMO–LUMO coupling is observed in the cases of C[DTBDT-BT]_4_ and C[DTBDT-DPP]_4_ when compared to other complexes leads to high charge transfer rates. The lowest charge recombination rate in L[PT]_12_─C[DTBDT-BT]_4_ results in the high *k*_CT_/*k*_CR_ ratio of 2.7 × 10^20^. Overall, the combination of L[PT]_12_─C[DTBDT-BT]_4_ and L[PT]_12_─C[DTBDT-DPP]_4_ has a stronger exciton dissociation and easier CT among all interfaces considered and thus can be considered as a promising combination of electron-donor and electron-acceptor materials.

**Table 7:** Calculated free energy change of charge transfer ΔG_CT_ (eV) and charge recombination ΔG_CR_ (eV), reorganization energy λ (eV), transfer integrals HOMO–HOMO coupling V_H-H_ (eV), LUMO–LUMO coupling V_L-L_ (eV), HOMO–LUMO coupling V_H-L_ (eV), rate of charge transfer (*k*_CT_) and charge recombination (*k*_CR_) in S^−1^ mPW1PW91/6-31G** level of theory.

D/A complex	ΔG_CT_	ΔG_CR_	λ	V_H-H_	V_L-L_	V_H-L_	*k*_CT_ hole	*k*_CT_ electron	*k*_CR_
L[PT]_12_/C[TT-DPP]_4_	− 0.53	− 1.58	0.51	0.002	0.002	0.02	9.34 × 10^11^	9.34 × 10^12^	3.09 × 10^3^
L[PT]_12_/C[BDT-DPP]_4_	− 0.70	− 1.41	0.46	0.003	0.031	0.05	6.59 × 10^10^	6.59 × 10^10^	3.19 × 10^5^
L[PT]_12_/C[DTBDT-DPP]_4_	− 0.53	− 1.59	0.45	0.004	0.076	0.02	3.49 × 10^11^	1.33 × 10^14^	6.31
L[PT]_12_/C[DTBDT-BT]_4_	− 0.27	− 1.84	0.44	0.005	0.099	0.01	3.34 × 10^11^	1.06 × 10^14^	3.8 × 10^–7^
L[PT]_12_/L[BDT-DPP]_4_	− 0.55	− 1.56	0.47	0.003	0.002	0.02	1.93 × 10^11^	6.20 × 10^10^	2.05 × 10^2^

## Conclusions

In conclusion, we have demonstrated the geometrical, electronic, and optical properties of new donor–acceptor-based conjugated macrocycle molecules using DFT and TD-DFT methodologies. The newly designed macromolecules show promising electronic properties due to radial π-conjugation. The choice of donor and acceptor units strongly influences the geometrical, electronic, and excited-state properties. The strain energy in the macrocyclic compounds decreases with the size of the ring. Introducing electron-rich and electron-poor units impacted the radial π-conjugation character along with the HOMO–LUMO energy level alteration. Designed molecules can be used as ambipolar as the hole and electron reorganization energies have marginal differences. Among all the designed macrocycles, large size compounds C[TT-DPP]_4_, C[BDT-DPP]_4_, C[DTBDT-DPP]_4_, and C[DTBDT-BT]_4_ can be used as electron acceptor materials for organic solar cell applications. The charge transfer integral values strongly depend on the packing configuration of dimers. Furthermore, we have also studied the interface properties between linear-cyclic and linear–linear complexes considering L[PT]_12_ as the donor component. Due to cyclization, the spatial distance between hole and electron in linear-cyclic complexes is doubled compared to the linear–linear complex, which can act as efficient materials for charge separation and can be potential molecules for organic solar cell applications. Based on various opto-electronic properties and charge transfer rate and charge recombination rate C[DTBDT-BT]_4_ shown better performance in electron transport and exciton separation.

## Materials and methods

The geometries of neutral and charged macrocyclic molecules and corresponding linear counterparts were optimized using density functional theory (DFT) based B3LYP/6-31G(d) method with dispersion correction. Further, single-point calculations were carried out to calculate optical and electronic properties using various DFT methods such as CAM-B3LYP and mPW1PW91 using the optimized geometries. In order to understand the strain in macrocyclic molecules, strain energies were calculated using B3LYP-D functional with the 6-31G(d,p) basis set. Excited-state analyses were performed using optimized geometries at TD-B3LYP/6-31G(d,p), TD-CAM-B3LYP/6-31G(d,p), and TD-mPW1PW91/6-31G(d,p) level of theory. Natural Transition Orbital (NTO) analysis was carried out to understand the nature of excited states^56^. All calculations were performed using the Gaussian16 package^57^.

The charge transport process in nonordered semiconductors can be explained using an incoherent hopping mechanism^40,46,58^. In the hopping mechanism, charge transfer occurs in sequential jumps between adjacent molecules^40,59,60^. Thus, the charge transfer rate can be described using Marcus theory. It is well known from the rate expression that the reorganization energy influences the rate of charge transfer^42,59,61–63^. Therefore, both hole and electron reorganization energies (λ_+_ and λ_−_) were calculated by using the following Eqs. (3) and (4)^47^.

3 $$\uplambda _{ + } = {\text{E}}^{ + } \left( {{\text{M}}^{0} } \right) - {\text{E}}^{ + } \left( {{\text{M}}^{ + } } \right) + {\text{E}}^{0} \left( {{\text{M}}^{ + } } \right) - {\text{E}}^{0} \left( {{\text{M}}^{0} } \right)$$

4 $$\uplambda _{ - } = {\text{E}}^{ - } \left( {{\text{M}}^{0} } \right) - {\text{E}}^{ - } \left( {{\text{M}}^{ - } } \right) + {\text{E}}^{0} \left( {{\text{M}}^{ + } } \right) - {\text{E}}^{0} \left( {{\text{M}}^{0} } \right)$$

Where E^0^_,_ E^+^, and E^−^ represents energy of neutral, cationic, and anion respectively and M^0^, M^+^, and M^−^ represents optimized geometries of neutral, cationic, and anion systems respectively.

The total strain energy (E_Strain_) in a macrocyclic compound is calculated using the following Eq. (5)^33,64^.

5 $${\text{E}}_{{{\text{Strain}}}} = \left( {{\text{E}}_{{{\text{Macrocycle}}}} + {\text{E}}_{{{\text{Cap}}}} } \right){-}{\text{E}}_{{{\text{Linear}}}}$$

At ambient temperature, the charge transport in organic solar cells is likely to occur through the thermally activated hopping model. In this model, the charge carriers localize on a single molecule and hop from one molecule to the adjacent molecule. The charge transfer rate constant (*k*) between equivalent neighbouring molecules can be defined by semiclassical Marcus theory. The charge transfer rate constant can be expressed as follows (the free energy difference (ΔG) for the self-exchange CT reaction process is neglected)^52,65,66^.

6 $$k = \frac{2\pi }{\hbar }V^{2} \left( {\frac{1}{{\sqrt {4\pi \lambda k_{B} T} }}} \right)\exp \left( { - \frac{\lambda }{{4k_{B} T}}} \right)$$

Where *k* is the rate of charge transfer for hole and electron (*k*_*h*_ and *k*_*e*_ respectively), V electronic coupling of the hole and electron transfer (V_h_ and V_e_) computed with the generalized Mulliken–Hush (GMH) method^44,67^. ħ is Planck’s constant, k_B_ is the Boltzmann constant, T is room temperature, and λ is the hole and electron reorganization energy (λ_h_ and λ_e_) of the charge transfer process calculated using Eqs. (3) and (4).

Further, to gain insights into impact of molecular modifications on the electron mobility (μ) in these newly designed NFAs, the Einstein–Smoluchowski equation is used estimate the drift mobility of hopping μ using given as follows^68,69^.

7 $$\mu = \frac{{\text{e}}}{{k_{B} T}}D$$

where μ is the mobility of hole and electron (μ_h_ and μ_e_), e is the electron charge, k_B_ is the Boltzmann constant, T is room temperature, and D is the diffusion coefficient which can be expressed in terms of the charge transfer rate constant *k* and r (the distance between two centroids of backbones of two molecules in one dimer) as follows:

8 $$D = \frac{1}{2}kr^{2}$$

Charge transfer at the donor–acceptor interface is the process by which an electron/hole of the donor/acceptor injects into the acceptor/donor after local excitation. Charge transfer at the interface in the modeled D/A systems can be roughly interpreted as electron/hole transfer driven by the donor/acceptor local excitation states. The rate of charge transfer (*k*_CT_) and charge recombination (*k*_CR_) in D/A systems can be evaluated by the Marcus theory as follows^65^.

9 $$k = \frac{2\pi }{\hbar }|V_{DA} \left. \right|^{2} \left( {\frac{1}{{\sqrt {4\pi \lambda k_{B} T} }}} \right)\exp \left( { - \frac{{\left( {\Delta G + \lambda } \right)^{2} }}{{4\lambda k_{B} T}}} \right)$$

Where *k* is the rate constant for charge transfer (*k*_CT_) and charge recombination (*k*_CR_), V_DA_ represents the CT integral between the donor and acceptor estimated using the GMH model, λ is the reorganization energy (internal and external), k_B_ is the Boltzmann constant, T is temperature, and ΔG is the free energy change for electron transfer process. The ΔG during the CT process is denoted as ΔG_CT_, and for the charge recombination process represented as ΔG_CR_.

The reorganization energy comprises two parts, inner reorganization energy (λ_i_) and external reorganization energy (λ_s_)^70–72^. The λ_i_ originates due to a change in the equilibrium geometry of the donor (D) and acceptor (A) sites of the complex system during charge transfer processes and it includes contribution of hole and electron which is formulated as follows:

10 $$\uplambda =\uplambda _{{\text{i}}} +\uplambda _{{\text{s}}}$$

11 $$\uplambda _{{\text{i}}} =\uplambda _{{\text{h}}} +\uplambda _{{\text{e}}}$$

12 $$\uplambda _{{\text{h}}} = {\text{E}}^{ + } \left( {{\text{D}}^{0} } \right) - {\text{E}}^{ + } \left( {{\text{D}}^{ + } } \right)$$

13 $$\uplambda _{{\text{e}}} = {\text{E}}^{0} \left( {{\text{A}}^{ - } } \right){-}{\text{E}}^{0} \left( {{\text{A}}^{0} } \right)$$

where E^+^(D^0^) and E^+^(D^+^) energies of radical cation donor at neutral geometry and optimal cation geometry. E^0^(A^−^) and E^0^(A^0^) are the energies of the neutral acceptor A at the anionic geometry and optimal ground state geometry, respectively. Calculating λ_s_ quantitatively is difficult as it involves electronic and nuclear polarizations. So, the value of λ_s_ is viewed as a constant equal to 0.3 eV.

The free energy change during charge recombination (ΔG_CR_) can be calculated as given below Eq.^71–73^

14 $$\Delta {\text{G}}_{{{\text{CR}}}} = {\text{IP}}\left( {\text{D}} \right){-}{\text{EA}}\left( {\text{A}} \right)$$

The IP(D) and EA(A) can be estimated as HOMO energy of donor and LUMO energy of acceptor, respectively. The free energy change at the CT process, ΔG_CT_, can be estimated using the Rehm–Weller equation as follows^73^.

15 $$\Delta {\text{G}}_{{{\text{CT}}}} = {-}\Delta {\text{G}}_{{{\text{CR}}}} {-}{\text{E}}_{{{\text{S}}1}} \left( {\text{D}} \right)$$

Where E_S1_(D) is the lowest excited-state energy of the free-base donor.

## Supplementary Information

Supplementary Information.

## Data Availability

The data that support the findings will be available from the authors if required.
