# Supplementary material for: Theoretical studies on donor–acceptor based macrocycles for organic solar cell applications
Source: Sci Rep. 2022 Sep 3;12:15043. doi: 10.1038/s41598-022-19348-5 (PMC9440932; doi:10.1038/s41598-022-19348-5)
Supplement: Supplementary file 1 — Supplementary Information. [file 41598_2022_19348_MOESM1_ESM.docx]

Theoretical Studies on Donor-Acceptor Based Macrocycles for Organic Solar Cell Applications

**Sheik Haseena^1^ and Mahesh Kumar Ravva^1^***

^1^Department of Chemistry, SRM University-AP, Andhra Pradesh, India 522240

*Corresponding author: [mahesh.r@srmap.edu.in](about:blank)

**Supporting Information**


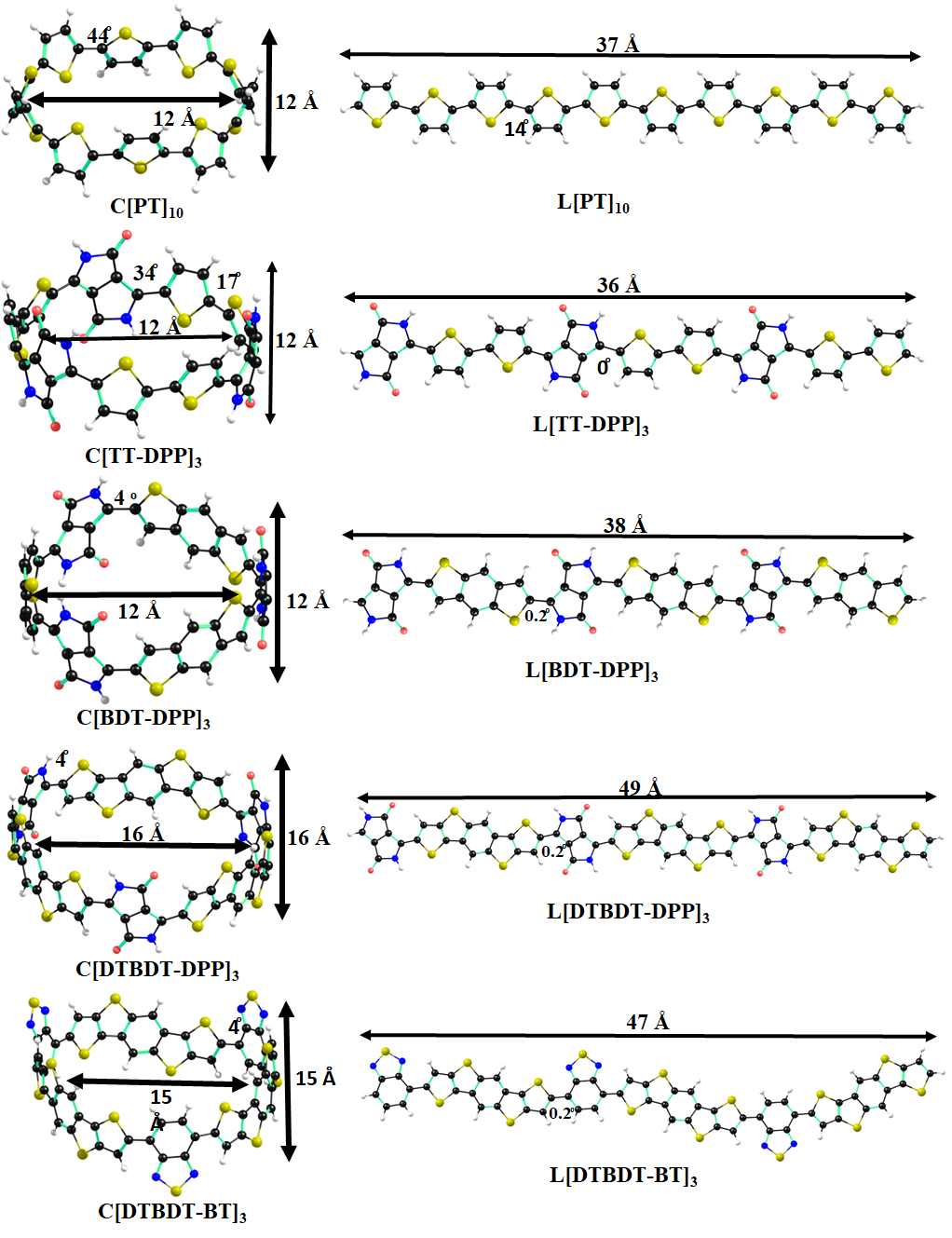


**Figure S1.** Optimized geometries of cyclic (C[PT]_10_, C[TT-DPP]_3_, C[BDT-DPP]_3_, C[DTBDT-DPP]_3,_ and C[DTBDT-BT]_3_) and linear (L[PT]_10_, L[TT-DPP]_3_, L[BDT-DPP]_3_, L[DTBDT-DPP]_3,_ and L[DTBDT-BT]_3_) donor-acceptor-based conjugated molecules as determined at B3LYP-D/6-31G(d,p) level of theory.

**
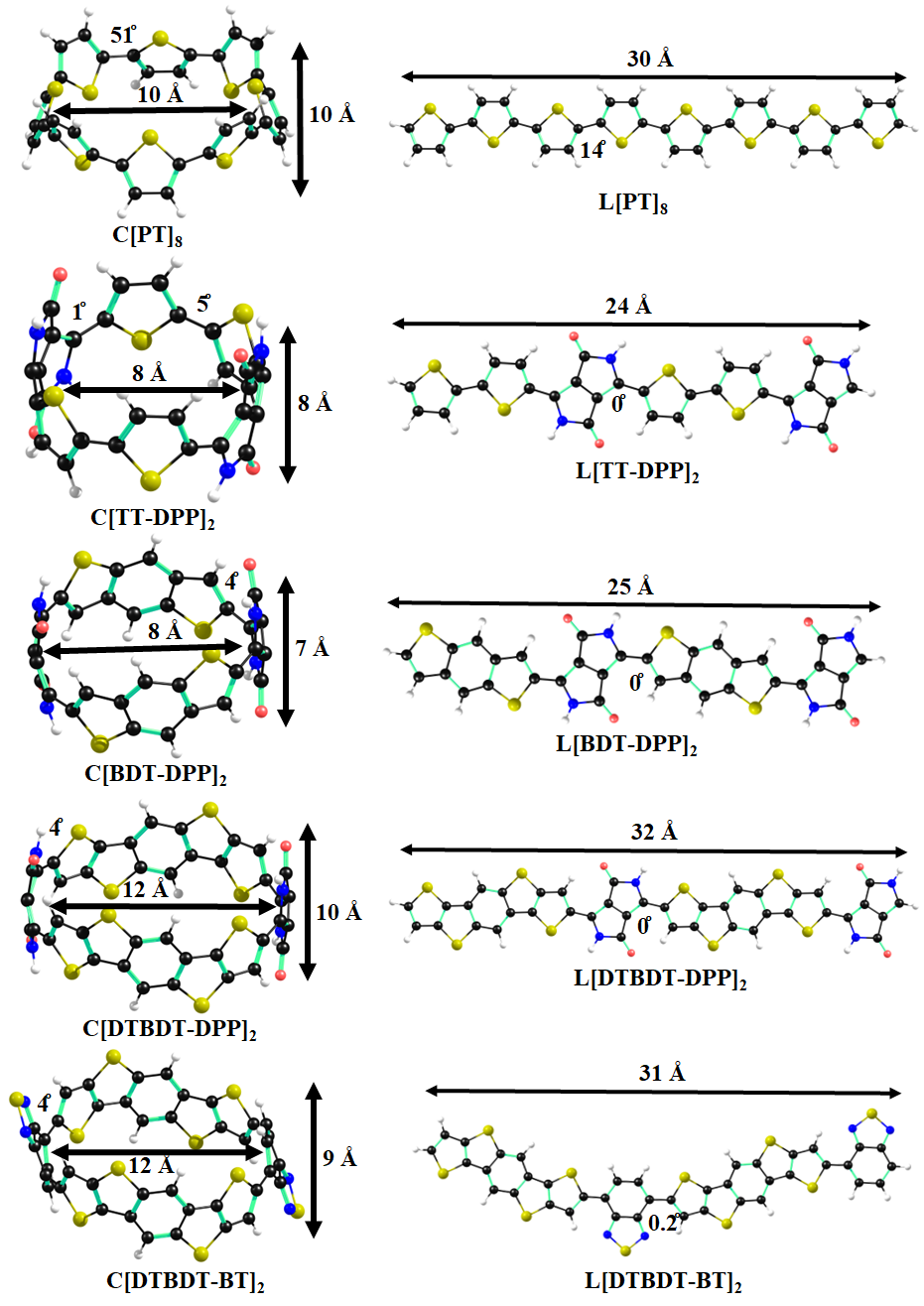
**

**Figure S2.** Optimized geometries of cyclic (C[PT]_8_, C[TT-DPP]_2_, C[BDT-DPP]_2_, C[DTBDT-DPP]_2,_ and C[DTBDT-BT]_2_) and linear (L[PT]_8_, L[TT-DPP]_2_, L[BDT-DPP]_2_, L[DTBDT-DPP]_2,_ and L[DTBDT-BT]_2_) donor-acceptor-based conjugated molecules as determined at B3LYP-D/6-31G(d,p) level of theory.


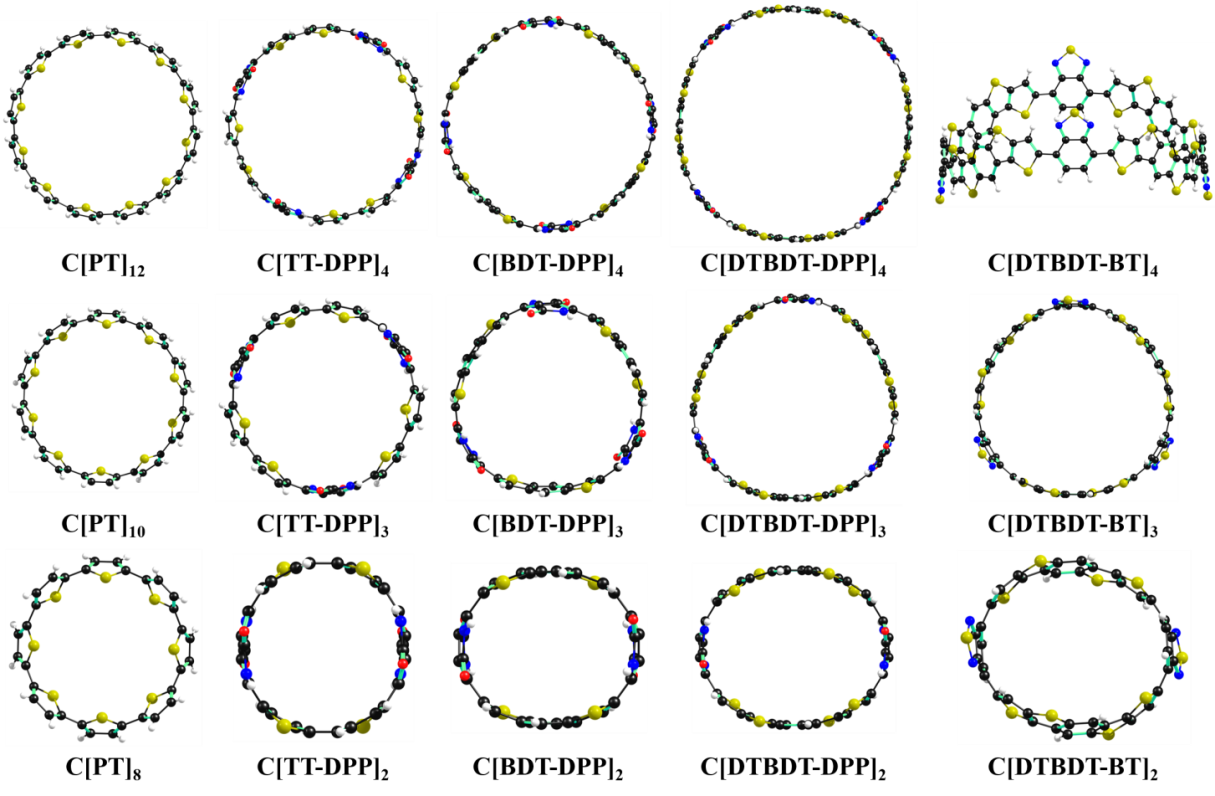


**Figure S3.** Shapes of conjugated macrocycles considered in this study.

^
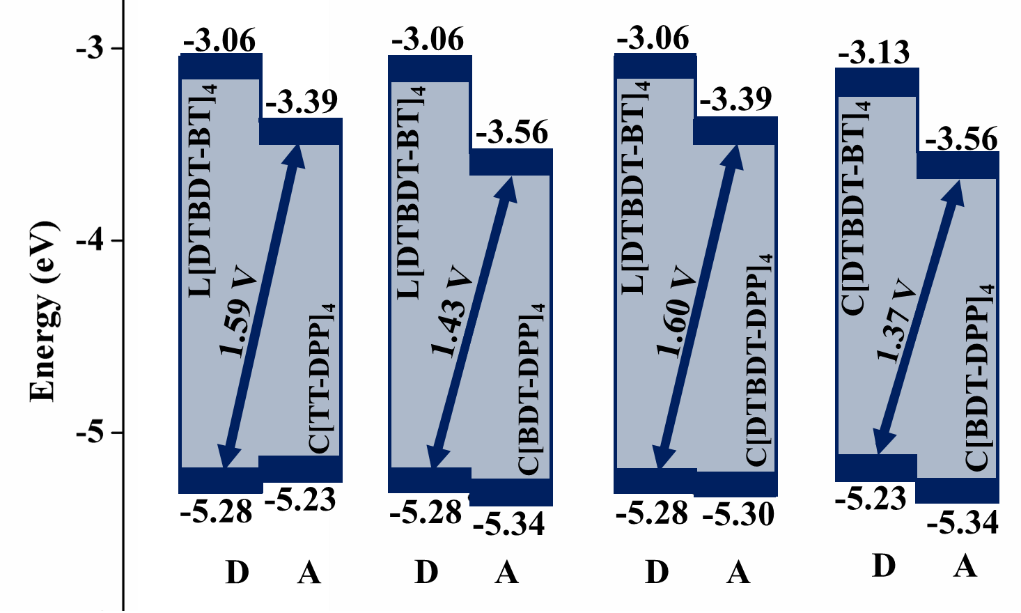
^

**Figure S4.** Open circuit voltage (V*oc*) of larger macrocyclic compounds with L[DTBDT-BT]_12_ and L[DTBDT-BT]_12_ as donor component.


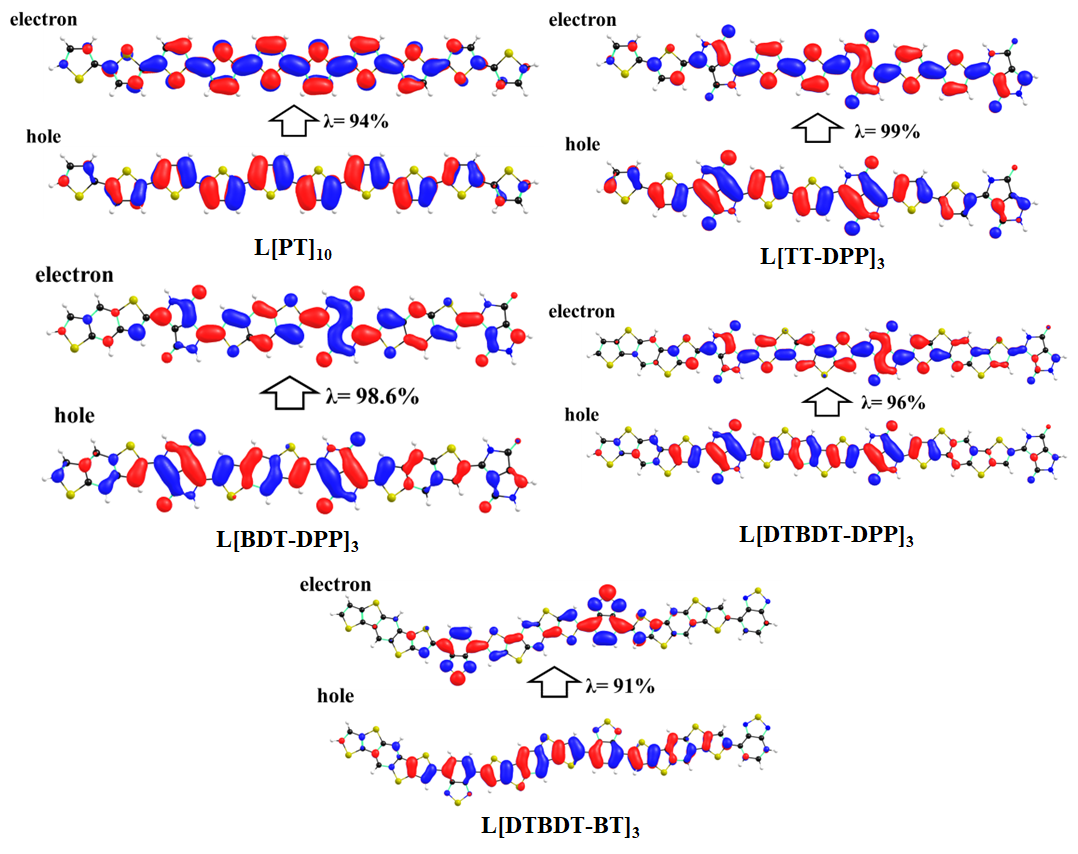


**Figure S5.** Pictorial representation of the natural transition orbitals of linear (L[PT]_10_, L[TT-DPP]_3_, L[BDT-DPP]_3_, L[DTBDT-DPP]_3,_ and L[DTBDT-BT]_3_) molecules corresponds to their lowest excited states (S_1_) calculated at TD-mpw1PW91/6-31G(d,p) level of theory. Where λ is the fraction of the hole–electron contribution to the excitation.


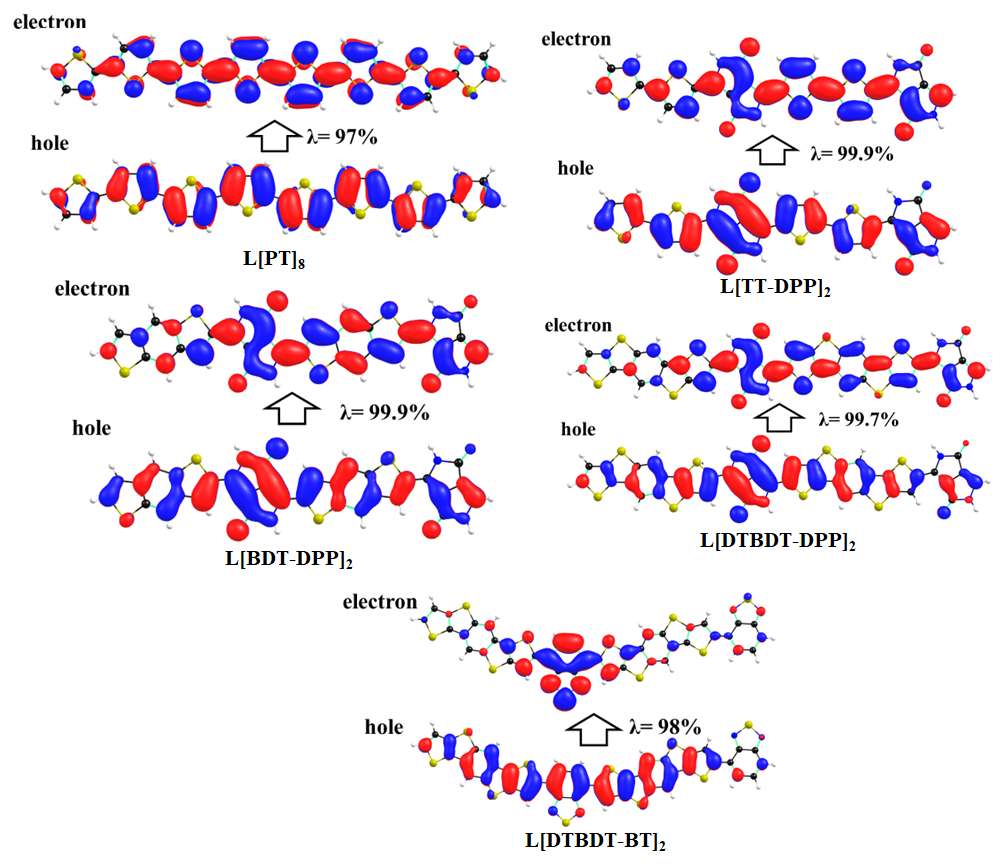


**Figure S6.** Pictorial representation of the natural transition orbitals of linear (L[PT]_8_, L[TT-DPP]_2_, L[BDT-DPP]_2_, L[DTBDT-DPP]_2,_ and L[DTBDT-BT]_2_) molecules corresponds to their lowest excited states (S_1_) calculated at TD-mPW1PW91/6-31G(d,p) level of theory. Where λ is the fraction of the hole–electron contribution to the excitation.


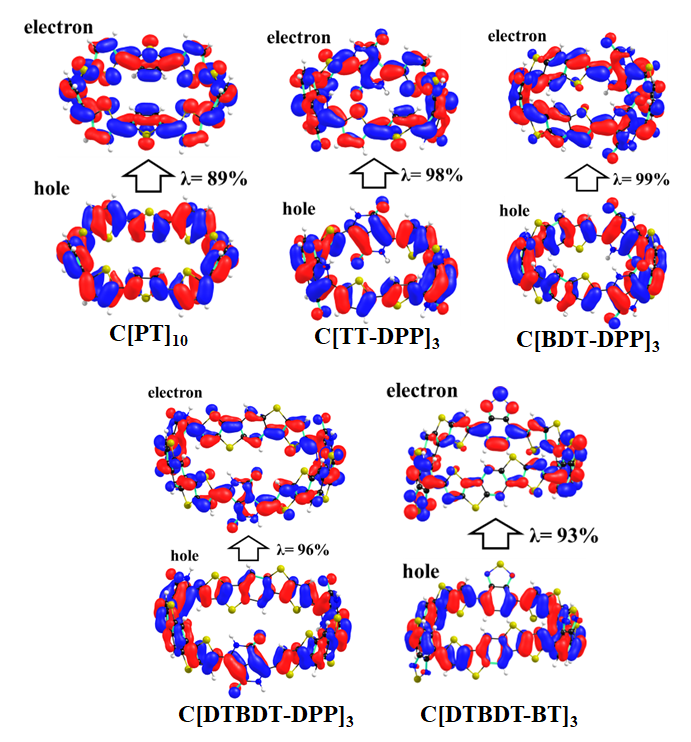


**Figure S7.** Pictorial representation of the natural transition orbitals of linear (C[PT]_10_, C[TT-DPP]_3_, C[BDT-DPP]_3_, C[DTBDT-DPP]_3,_ and C[DTBDT-BT]_3_) molecules corresponds to their lowest excited states (S_1_) calculated at TD-mpw1PW91/6-31G(d,p) level of theory. Where λ is the fraction of the hole–electron contribution to the excitation.


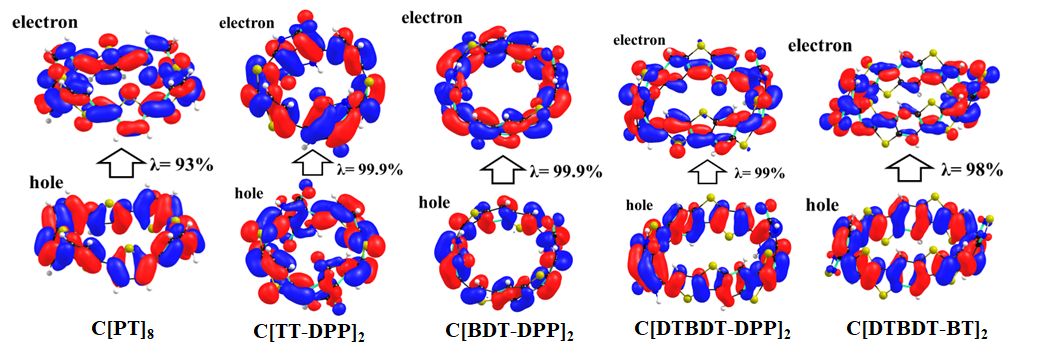


**Figure S8.** Pictorial representation of the natural transition orbitals of linear (C[PT]_8_, C[TT-DPP]_2_, C[BDT-DPP]_2_, C[DTBDT-DPP]_2,_ and C[DTBDT-BT]_2_) molecules corresponds to their lowest excited states (S_1_) calculated at TD-mpw1PW91/6-31G(d,p) level of theory. Where λ is the fraction of the hole–electron contribution to the excitation.

**
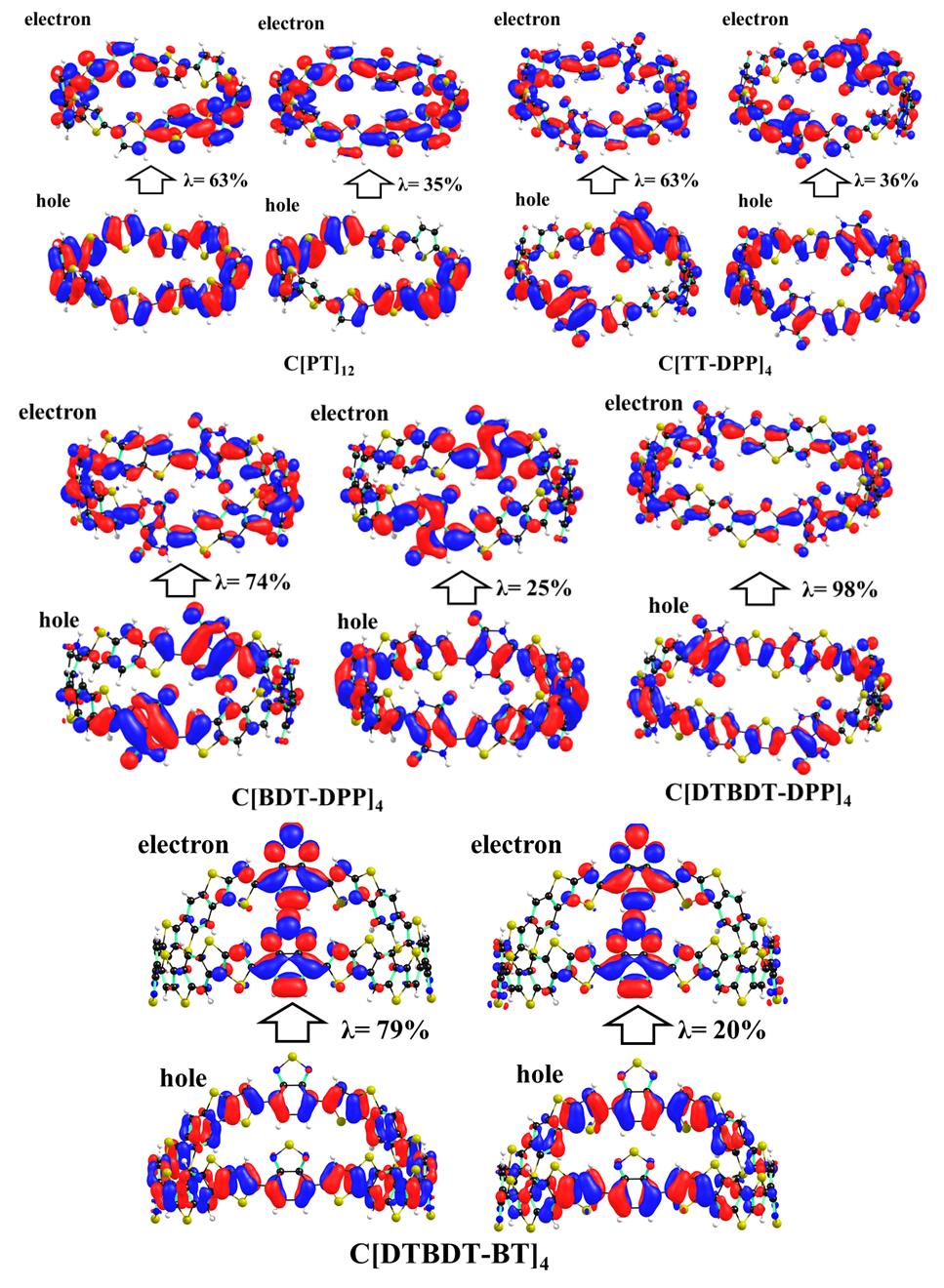
**

**Figure S9.** Pictorial representation of the natural transition orbitals at second lowest excited state S_2_ in the cases of C[PT]_12_, C[DTBDT-DPP]_4_ and C[DTBDT-BT]_4_ and at S_4_ for C[TT-DPP]_4_ and C[BDT-DPP]_4_ calculated at TD-mPW1PW91/6-31G(d,p) level of theory. Where, λ is the fraction of the hole–electron contribution to the excitation.

| 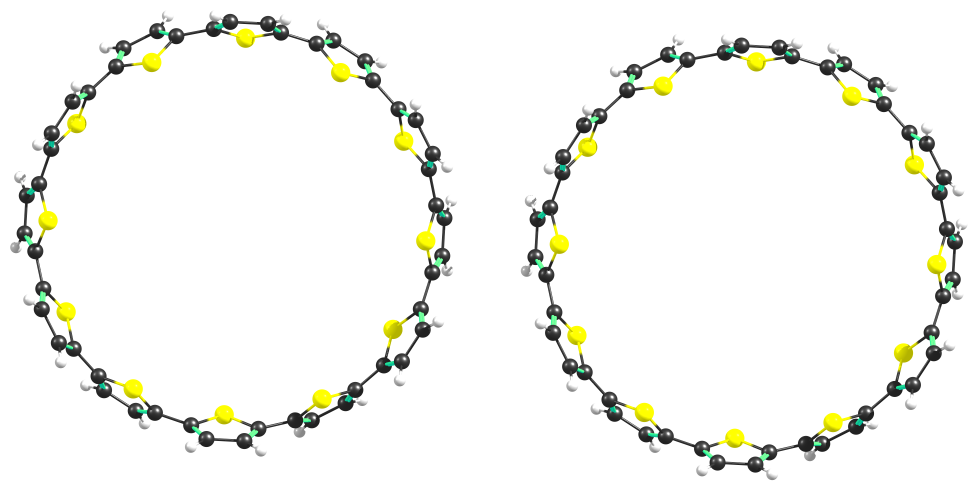 | | |
| --- | --- | --- |
| C[PT]_12_ – DD | | |
| 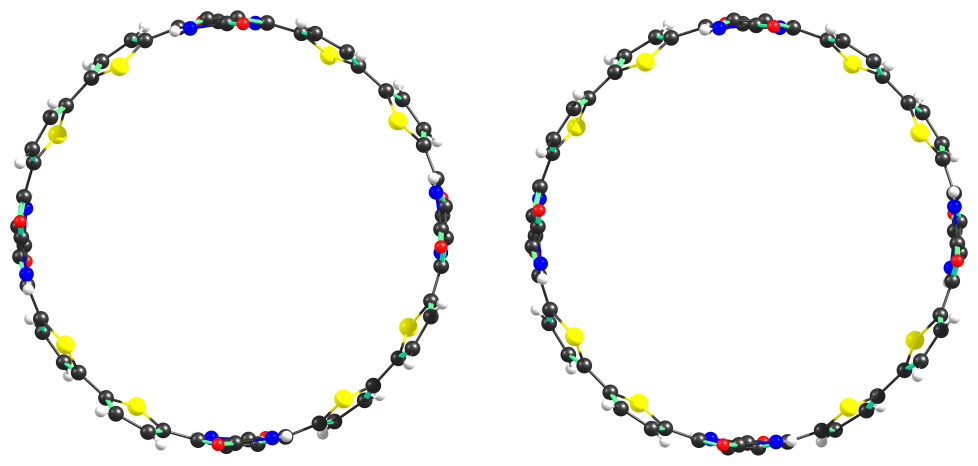 | 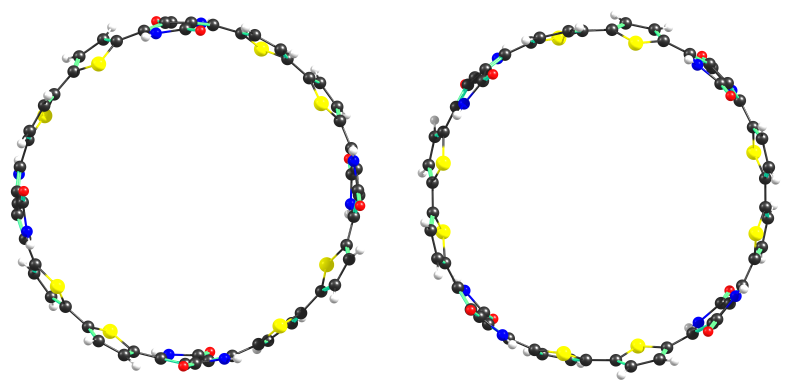 | 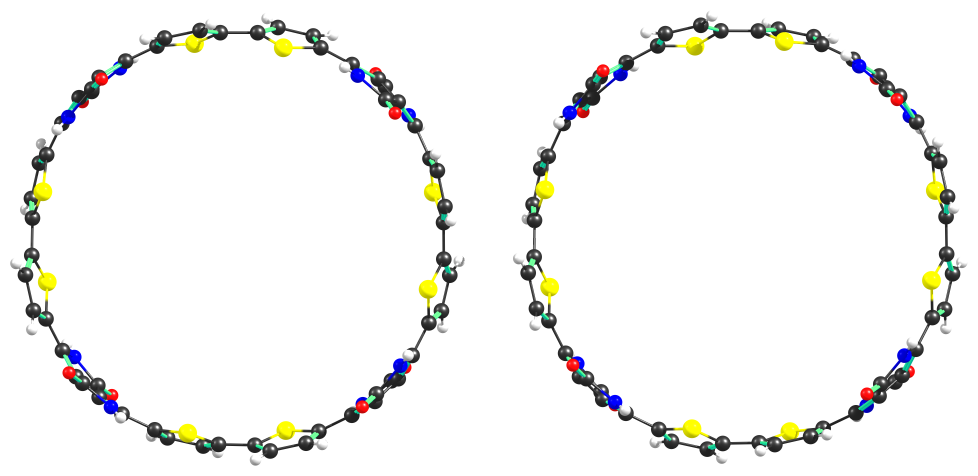 |
| C[TT-DPP]_4_ - AA | C[TT-DPP]_4_ – DA | C[TT-DPP]_4_ – DD |
| 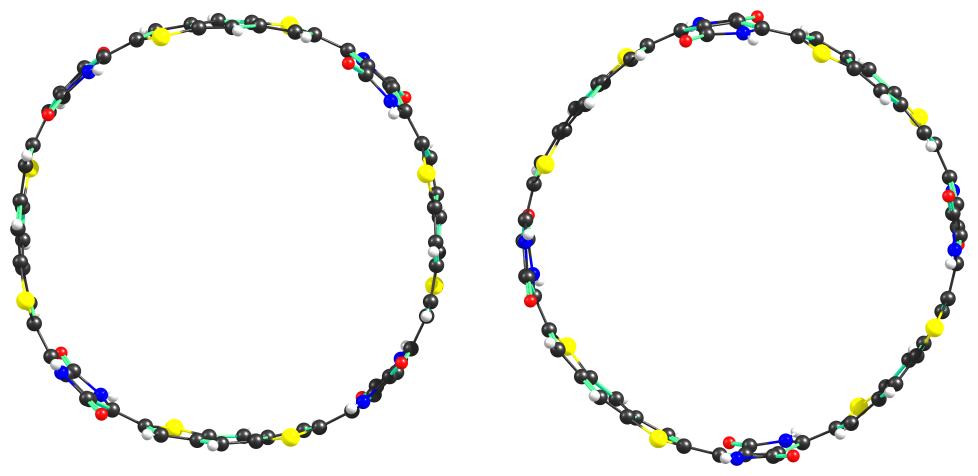 | 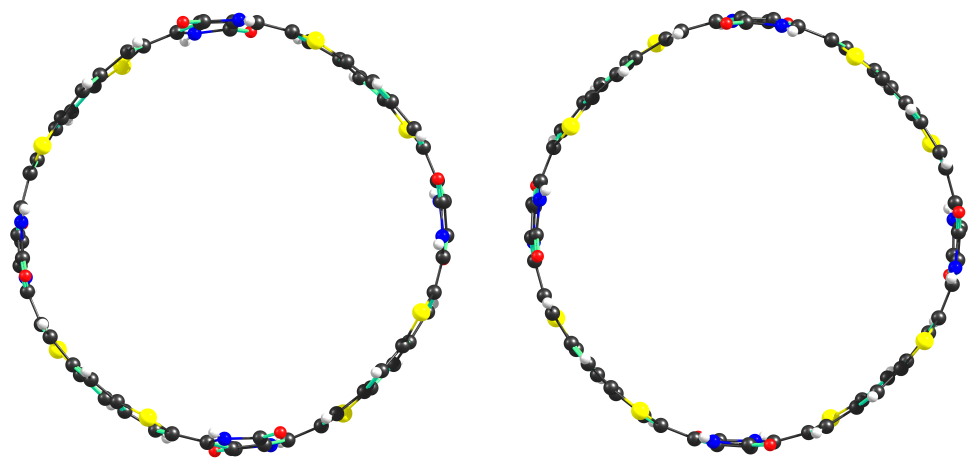 | 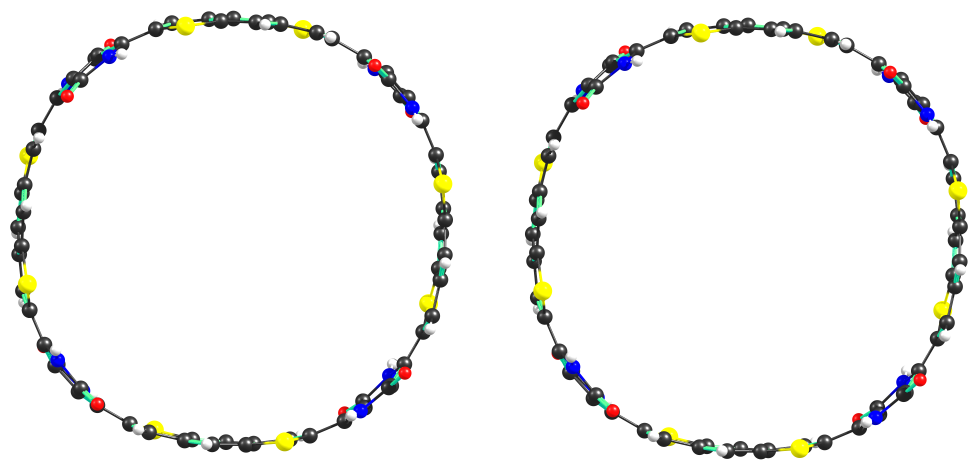 |
| C[BDT-DPP]_4_ - AA | C[BDT-DPP]_4_ – DA | C[BDT-DPP]_4_ – DD |
| 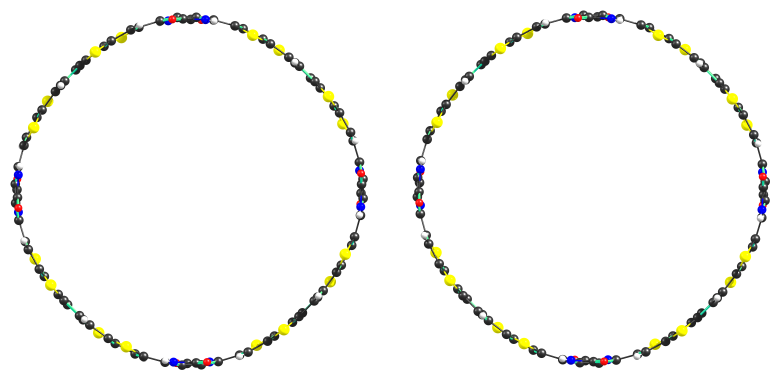 | 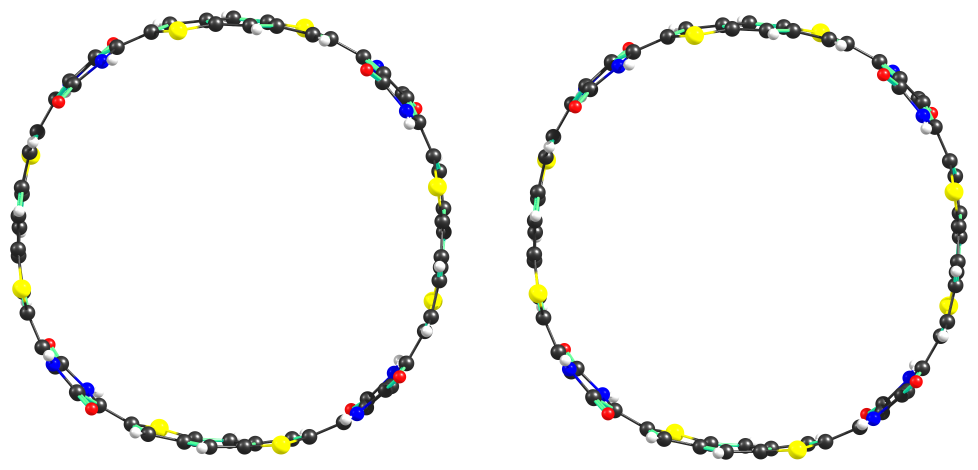 | 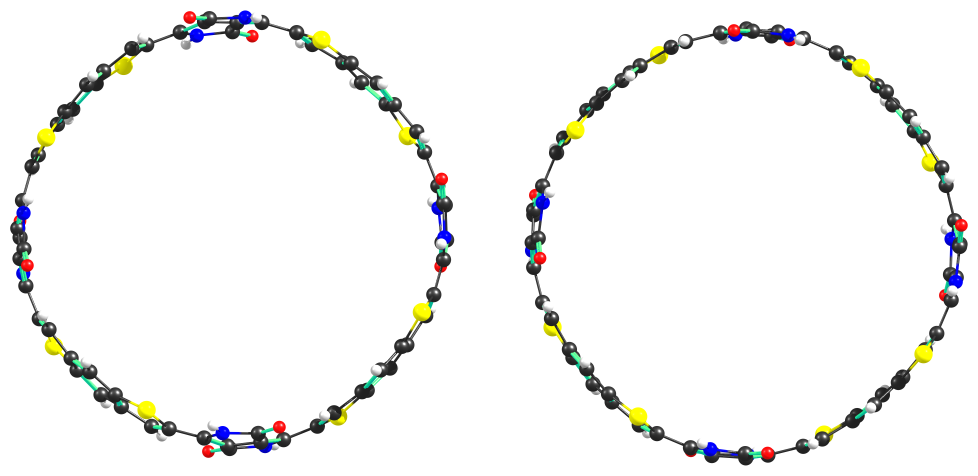 |
| C[DTBDT-DPP]_4_ - AA | C[DTBDT-DPP]_4_ – DA | C[DTBDT-DPP]_4_ – DD |
| 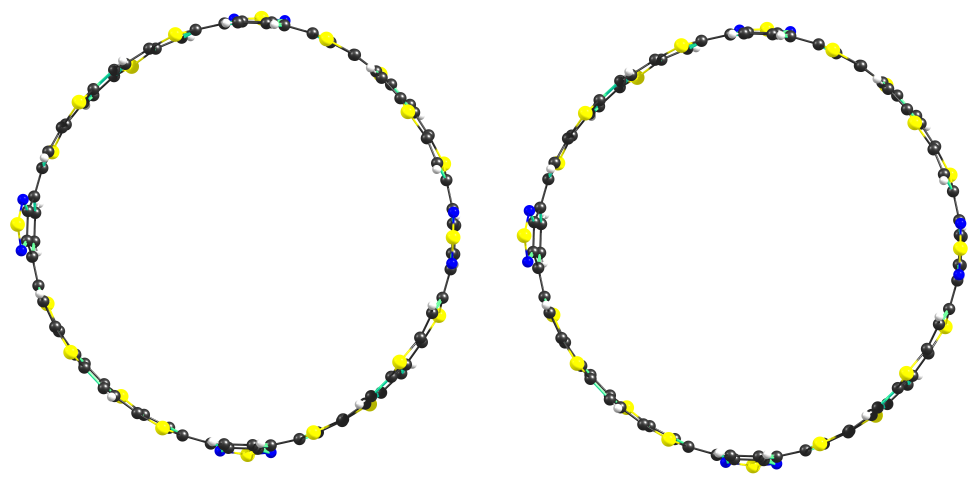 | 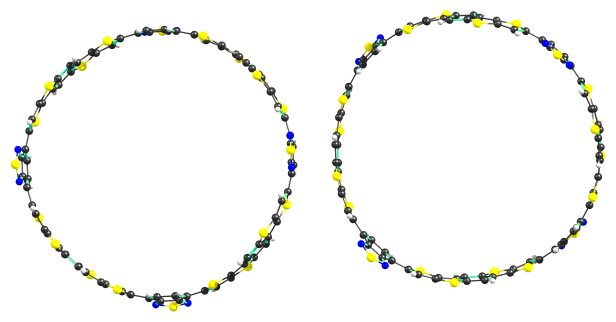 | 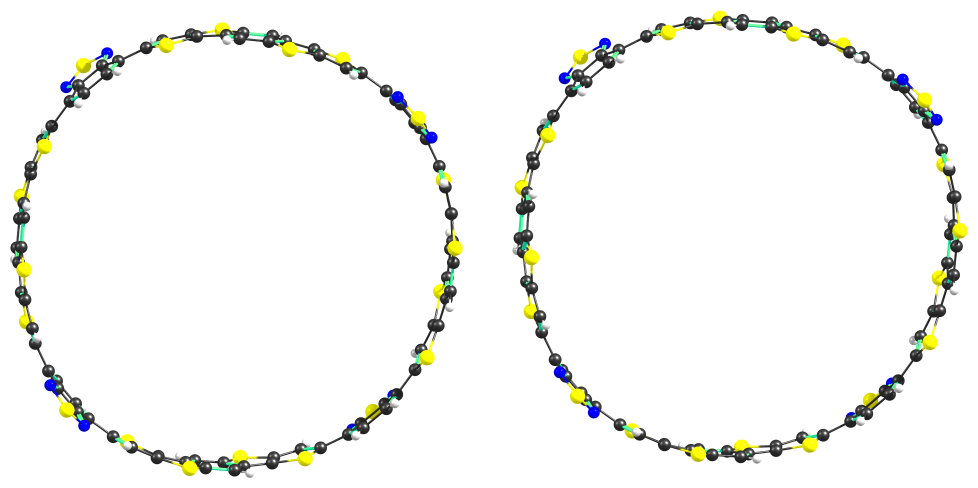 |
| C[DTBDT-BT]_4_ - AA | C[DTBDT-BT]_4_ – DA | C[DTBDT-BT]_4_ – DD |

**Figure S10.** Dimer configurations of various cyclic macrocycles used for charge transfer integrals calculations. In each dimer case, three types of interactions, viz., the donor unit of one ring interacts with the donor unit of other ring (DD), the donor unit of one ring interacts with the acceptor unit of other ring (DA), and the acceptor unit of one ring interacts with the acceptor unit of other ring (AA) in C[PT]_12_, C[TT-DPP]_4_, C[BDT-DPP]_4_, C[DTBDT-DPP]_4,_ and C[DTBDT-BT]_4_ complexes. The distance between the rings are fixed at 3.5 Å in all complexes.

|  | B3LYP | | CAM-B3LYP | | mPW1PW91 | | Exp | |
| --- | --- | --- | --- | --- | --- | --- | --- | --- |
|  | HOMO | LUMO | HOMO | LUMO | HOMO | LUMO | HOMO | LUMO |
| C[TT-DPP]_4_ | -4.96 | -3.36 | -6.06 | -2.46 | -5.23 | -3.39 | -5.33 | -3.44 |
| L[TT-DPP]_4_ | -4.94 | -3.38 | -5.97 | -2.56 | -5.16 | -3.45 | -5.11 | -3.66 |

**Table S1.** Calculated HOMO and LUMO energies for various conjugated macrocycles considered in the study using different functionals with 6-31G** level of theory. Experimental values are also given for comparison. All values are in eV.

|  | HOMO | LUMO | E_g_ | LUMO+1 | ΔE_LUMO+1-LUMO_ |
| --- | --- | --- | --- | --- | --- |
| C[PT]_8_ | -5.43 | -1.61 | 3.82 | -1.19 | 0.42 |
| C[TT-DPP]_2_ | -5.08 | -3.56 | 1.52 | -2.69 | 0.86 |
| C[BDT-DPP]_2_ | -5.39 | -3.51 | 1.87 | -2.55 | 0.96 |
| C[DTBDT-DPP]_2_ | -5.32 | -3.32 | 2.00 | -2.72 | 0.60 |
| C[DTBDT-BT]_2_ | -5.17 | -3.14 | 2.03 | -2.79 | 0.35 |
|  |  |  |  |  |  |
| C[PT]_10_ | -5.28 | -1.83 | 3.46 | -1.47 | 0.36 |
| C[TT-DPP]_3_ | -5.30 | -3.24 | 2.05 | -2.69 | 0.56 |
| C[BDT-DPP]_3_ | -5.35 | -3.55 | 1.81 | -2.88 | 0.67 |
| C[DTBDT-DPP]_3_ | -5.30 | -3.37 | 1.94 | -2.95 | 0.42 |
| C[DTBDT-BT]_3_ | -5.19 | -3.13 | 2.06 | -2.88 | 0.25 |
|  |  |  |  |  |  |
| C[PT]_12_ | -5.19 | -1.97 | 3.21 | -1.68 | 0.29 |
| C[TT-DPP]_4_ | -5.23 | -3.39 | 1.84 | -2.98 | 0.41 |
| C[BDT-DPP]_4_ | -5.34 | -3.56 | 1.78 | -3.12 | 0.44 |
| C[DTBDT-DPP]_4_ | -5.30 | -3.39 | 1.91 | -3.11 | 0.27 |
| C[DTBDT-BT]_4_ | -5.23 | -3.13 | 2.10 | -2.95 | 0.17 |
|  |  |  |  |  |  |
| L[PT]_8_ | -5.04 | -2.25 | 2.79 | -1.80 | 0.45 |
| L[TT-DPP]_2_ | -5.43 | -3.15 | 2.28 | -2.54 | 0.61 |
| L[BDT-DPP]_2_ | -5.28 | -3.16 | 2.11 | -2.50 | 0.66 |
| L[DTBDT-DPP]_2_ | -5.37 | -3.12 | 2.25 | -2.68 | 0.44 |
| L[DTBDT-BT]_2_ | -5.37 | -2.94 | 2.44 | -2.64 | 0.30 |
|  |  |  |  |  |  |
| L[PT]_10_ | -5.00 | -2.33 | 2.66 | -1.99 | 0.34 |
| L[TT-DPP]_3_ | -5.20 | -3.36 | 1.84 | -2.92 | 0.44 |
| L[BDT-DPP]_3_ | -5.30 | -3.03 | 2.28 | -2.86 | 0.16 |
| L[DTBDT-DPP]_3_ | -5.32 | -3.25 | 2.06 | -2.97 | 0.29 |
| L[DTBDT-BT]_3_ | -5.30 | -3.03 | 2.28 | -2.86 | 0.16 |
|  |  |  |  |  |  |
| L[PT]_12_ | -4.97 | -2.38 | 2.60 | -2.12 | 0.26 |
| L[TT-DPP]_4_ | -5.16 | -3.45 | 1.71 | -3.14 | 0.31 |
| L[BDT-DPP]_4_ | -5.33 | -3.41 | 1.92 | -3.13 | 0.29 |
| L[DTBDT-DPP]_4_ | -5.30 | -3.31 | 1.99 | -3.12 | 0.19 |
| L[DTBDT-BT]_4_ | -5.28 | -3.06 | 2.22 | -2.95 | 0.12 |

**Table S2.** Calculated HOMO, LUMO, LUMO+1 energies for various conjugated macrocycles considered in the study at the mPW1PW91/6-31G** level of theory. The energy difference between HOMO, LUMO energies (E_g_), LUMO, LUMO+1 (ΔE_LUMO+1-LUMO_) also given. All values are in eV.

| Donor | Acceptor | L_D_-L_A_ | V*oc* |
| --- | --- | --- | --- |
| L[PT]_12_ | C[TT-DPP]_4_ | 1.01 | 1.28 |
| L[PT]_12_ | C[BDT-DPP]_4_ | 1.18 | 1.11 |
| L[PT]_12_ | C[DTBDT-DPP]_4_ | 1.01 | 1.29 |
| L[PT]_12_ | C[DTBDT-BT]_4_ | 0.75 | 1.54 |
|  |  |  |  |
| C[PT]_12_ | C[TT-DPP]_4_ | 1.42 | 1.50 |
| C[PT]_12_ | C[BDT-DPP]_4_ | 1.59 | 1.33 |
| C[PT]_12_ | C[DTBDT-DPP]_4_ | 1.42 | 1.50 |
| C[PT]_12_ | C[DTBDT-BT]_4_ | 1.16 | 1.76 |
|  |  |  |  |
| L[DTBDT-BT]_4_ | C[TT-DPP]_4_ | 0.33 | 1.59 |
| L[DTBDT-BT]_4_ | C[BDT-DPP]_4_ | 0.49 | 1.43 |
| L[DTBDT-BT]_4_ | C[DTBDT-DPP]_4_ | 0.32 | 1.60 |
| C[DTBDT-BT]_4_ | C[BDT-DPP]_4_ | 0.43 | 1.37 |

**Table S3.** Calculated energetic driving force (L_D_-L_A_) in eV and V*oc* (V) at mPW1PW91/6-31G** level of theory.

|  | S_2_ | f | S_3_ | f | S_4_ | f | S_5_ | f |
| --- | --- | --- | --- | --- | --- | --- | --- | --- |
| C[PT]_12_ | 407.77 | 1.82 | 407.77 | 1.82 |  |  |  |  |
| C[TT-DPP]_4_ |  |  |  |  | 608.12 | 1.90 | 608.10 | 1.90 |
| C[BDT-DPP]_4_ |  |  |  |  | 620.54 | 1.74 | 620.54 | 1.74 |
| C[DTBDT-DPP]_4_ | 645.67 | 1.08 | 645.67 | 1.08 | 637.74 | 1.67 | 637.73 | 1.67 |
| C[DTBDT-BT]_4_ | 641.09 | 1.60 | 641.01 | 1.60 |  |  |  |  |
|  |  |  |  |  |  |  |  |  |
| C[PT]_10_ | 373.79 | 1.26 | 373.78 | 1.26 |  |  |  |  |
| C[TT-DPP]_3_ |  |  |  |  | 522.51 | 1.10 |  |  |
| C[BDT-DPP]_3_ |  |  |  |  |  |  | 553.15 | 1.04 |
| C[DTBDT-DPP]_3_ |  |  |  |  | 580.02 | 1.75 |  |  |
| C[DTBDT-BT]_3_ | 629.86 | 0.59 |  |  |  |  |  |  |
|  |  |  |  |  |  |  |  |  |
| C[PT]_8_ | 337.75 | 0.64 |  |  |  |  |  |  |
| C[TT-DPP]_2_ |  |  |  |  | 467.06 | 0.77 |  |  |
| C[BDT-DPP]_2_ |  |  |  |  |  |  |  |  |
| C[DTBDT-DPP]_2_ |  |  | 519.51 | 0.89 |  |  |  |  |
| C[DTBDT-BT]_2_ |  |  |  |  |  |  |  |  |

**Table S4.** Excited state energies (S_2_ toS_5_) in nm with bright oscillator strength.
